# Recent Progress in the Development of Selective MAGL Modulators (2020–2026)

**DOI:** 10.3390/molecules31132353

**Published:** 2026-07-03

**Authors:** Eva Landucci, Chiara Lonzi, Tommaso Bonomo, Simone Bertini, Marco Macchia, Carlotta Granchi, Giulia Bononi

**Affiliations:** 1 Department of Pharmacy, University of Pisa, Via Bonanno 6, 56126 Pisa, Italy; e.landucci1@student.unisi.it (E.L.); chiara.lonzi@phd.unipi.it (C.L.); t.bonomo2@studenti.unipi.it (T.B.); simone.bertini@unipi.it (S.B.); marco.macchia@unipi.it (M.M.); giulia.bononi@unipi.it (G.B.); 2 Department of Life Sciences, University of Siena, Via Aldo Moro 2, 53100 Siena, Italy

**Keywords:** monoacylglycerol lipase, MAGL, MAGL modulators, MAGL inhibitors, anti-MAGL PROTACs, MAGL degraders

## Abstract

Monoacylglycerol lipase (MAGL) is a key enzyme at the interface between the endocannabinoid system and lipid metabolism, playing a pivotal role in the hydrolysis of the endocannabinoid 2-arachidonoylglycerol and in the regulation of lipid mediators involved in inflammation, pain, neurodegeneration and cancer. Owing to its therapeutic relevance, MAGL has emerged as an attractive pharmacological target, stimulating extensive research efforts aimed at the development of potent and selective modulators of its activity. Advances in medicinal chemistry, together with the increasing application of innovative computational approaches and biochemical methods to assess MAGL activity, have significantly expanded the chemical space of compounds capable of modulating this enzyme. This review provides a comprehensive overview of selective MAGL modulators reported in the scientific literature from 2020 to the present, excluding compounds described exclusively in patent literature and MAGL probes, as this area has been recently reviewed elsewhere, ranging from classical enzyme inhibitors to modulators acting through alternative strategies, such as targeted protein degradation. Overall, this review highlights the structural diversity and the main strategies that have emerged in recent years in modulating MAGL and it aims to guide the rational design of next-generation MAGL-targeting agents.

## 1. Introduction

The endocannabinoid system (ECS) is a widespread modulatory network that regulates numerous physiological processes throughout the body. The ECS was identified through research on bioactive compounds found in *Cannabis sativa*, from which the system takes its name. Studies of these compounds, including Δ-9-tetrahydrocannabinol (THC), led researchers to hypothesize the existence of specific cannabinoid receptors, ultimately resulting in the discovery of the ECS [1,2]. The system is primarily composed of two G-protein coupled receptors, cannabinoid receptor 1 (CB1) and 2 (CB2), their endogenous ligands known as endocannabinoids and the enzymes responsible for maintaining system homeostasis. CB1 and CB2 receptors differ in both amino acidic sequence and tissue distribution [3]. CB1 receptors are predominantly expressed in the central nervous system (CNS), especially in regions involved in cognitive and physiological functions, such as the hippocampus, cerebral cortex, cerebellum, basal ganglia, hypothalamus and amygdala. They are mainly localized in presynaptic terminals, where they regulate synaptic transmission. CB1 receptors are also present in glial cells including astrocytes, oligodendrocytes and microglia, as well as in peripheral tissues. In the gastrointestinal tract, for example, they play a role in controlling motility, gastric acid secretion and epithelial permeability. CB2 receptors share approximately 44% sequence homology with CB1 receptors and are primarily expressed in immune cells, although they can also be expressed in neurons, especially in pathological states [4]. They contribute to the analgesic and antinociceptive effects associated with cannabinoids [3]. The best-characterized endocannabinoids are anandamide (AEA) and 2-arachidonoylglycerol (2-AG). Unlike classical neurotransmitters, endocannabinoids are not stored in vesicles, instead, they are synthesized on demand from lipid membrane precursors and released into the extracellular space, where they act through a retrograde signaling mechanism [5]. Functionally, 2-AG acts as a high-efficacy agonist at both CB1 and CB2 receptors, whereas anandamide is a low-efficacy agonist at CB1 and exhibits very low efficacy at CB2. Endocannabinoid signaling is tightly regulated by enzymes involved in both synthesis and degradation. Focusing on 2-AG degradation, this endocannabinoid is primarily hydrolyzed by monoacylglycerol lipase (MAGL), producing arachidonic acid (AA) and glycerol. Additional enzymes, including α/β hydrolase domain-containing proteins 6 (ABHD6) and 12 (ABHD12), also contribute to its metabolism [6]. In particular, MAGL is responsible for approximately 85% of 2-AG hydrolysis in the brain [6], making it a major regulator of endocannabinoid signaling.

### 1.1. MAGL: Structural Features and Catalytic Mechanism

**Figure 1:**
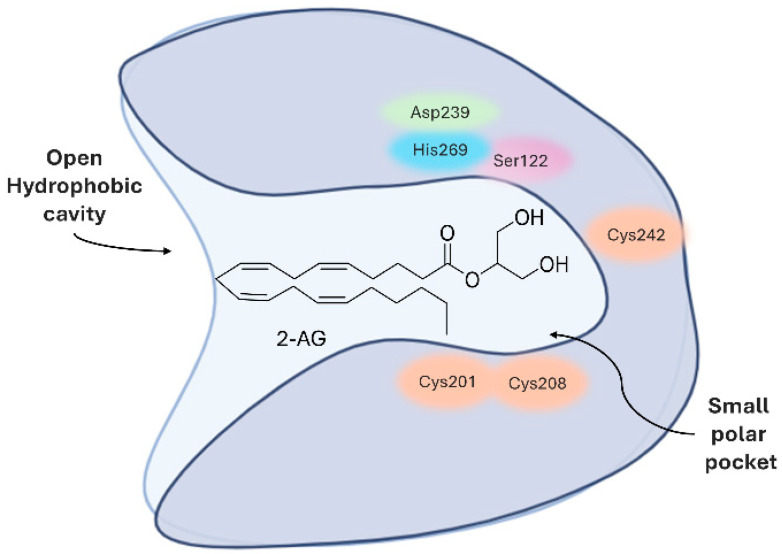
Representation of MAGL catalytic site.

Monoacylglycerol lipase (MAGL) is a α/β hydrolase that belongs to the serine hydrolase superfamily and plays a key role in both lipid metabolism and regulation of the ECS. This enzyme preferentially hydrolyzes monoacylglycerols (MAGs), rather than di- or triglycerides, generating glycerol and free fatty acids (FFAs). MAGL was first fully purified in 1976 from rat adipose tissue [7]. The initial determination of its primary structure was carried out in mice, where the enzyme was identified as a protein composed of 302 amino acids with a molecular weight of approximately 33.2 kDa [8]. Subsequent studies characterized the rat and human forms, both consisting of 303 amino acids and having a molecular weight of about 33.4 kDa. Like other members of the serine hydrolase family, MAGL contains a catalytic triad composed of Ser, Asp, and His residues. In human MAGL (*h*MAGL), these correspond to Ser122, Asp239 and His269, as confirmed by site-directed mutagenesis (Figure 1) [9,10]. The three-dimensional structure of *h*MAGL has been resolved by X-ray crystallography at a resolution of 2.2 Å. Structural analyses describe the enzyme as a dimer, with the two monomers forming an interface of approximately 884 Å^2^. This dimeric organization is supported by mass spectrometry data, which does not show evidence of a monomeric form [11]. From a structural point of view, MAGL is characterized by two main elements: the α/β hydrolase fold and the lid domain. The α/β fold consists of a central β-sheet made up of seven parallel strands and one antiparallel strand, surrounded by eight α-helices [12]. The catalytic serine is located within a conserved Gly-His-Ser-Met-Gly sequence, a typical feature of this enzyme family. This motif, often referred to as the “nucleophilic elbow”, forms a sharp γ-turn that positions the serine residue correctly for catalysis, allowing optimal interaction with the histidine residue and the substrate [13]. The lid domain represents another important structural feature, as it facilitates substrate access to the active site. It is positioned at the entrance of a large hydrophobic tunnel that extends toward the catalytic serine [14]. This tunnel, which ends in a more polar region, is well suited to accommodate the structure of 2-AG. It also connects to the solvent through an opening at the top and a smaller channel near the catalytic site, enabling substrate entry and product release. In addition, three cysteine residues (Cys201, Cys208 and Cys242) positioned close to the catalytic triad contribute to the stabilization of the enzyme’s active conformation. Their presence also offers a strategic advantage for the design of selective MAGL inhibitors, with more specificity to MAGL compared to other serine hydrolases [15]. Another important region of *h*MAGL is the “oxyanion hole”, constituted by the backbone NH group of Ala51 and Met123, that stabilizes the intermediate formed during the hydrolysis process of the substrate.

The catalytic mechanism of MAGL involves two main stages: substrate binding followed by hydrolysis [16]. Once the substrate enters the active site, the catalytic triad initiates the reaction. His269 acts as a base to deprotonate and Ser122 for nucleophilic attack, while Asp239 stabilizes and enhances the basicity of His269. This enables Ser122 to cleave the ester bond of 2-AG, forming a transient intermediate that is subsequently degraded, releasing glycerol and AA, while restoring the enzyme to its original state for further catalytic cycles.

### 1.2. MAGL as a Therapeutic Target

The central role of MAGL in regulating 2-AG levels, together with the wide range of physiological processes in which this endocannabinoid is involved, has made MAGL a major target for drug development. Notably, endogenous levels of 2-AG are substantially higher than those of AEA [17], further emphasizing its biological relevance. 2-AG is involved in several key functions, including pain perception, neuroprotection and appetite regulation [18,19,20,21].

**Figure 2:**
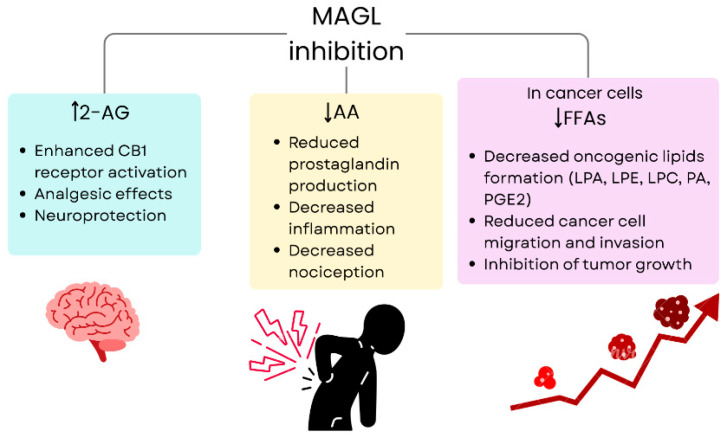
Representation of the main therapeutic effects of MAGL inhibition.

Although multiple enzymes contribute to its metabolism, MAGL is the primary enzyme responsible for 2-AG hydrolysis into AA and glycerol. This is important because AA can then be further processed by enzymes such as cyclooxygenases (COX-1 and COX-2) into pro-inflammatory mediators, including prostaglandins and thromboxanes. Accordingly, MAGL inhibition can relieve pain through a dual mechanism: involving both enhancement of endocannabinoid signaling and reduction in pro-inflammatory mediators. Since cannabinoid-induced analgesia is primarily mediated by CB1 receptor activation, the elevation of 2-AG following MAGL inhibition is considered a key contributor to the resulting antinociceptive effects. Moreover, inhibition of MAGL simultaneously decreases the formation of AA-derived pronociceptive mediators [22]. MAGL inhibition also contributes to neuroprotective effects; firstly, it increases brain 2-AG levels, which has neuroprotective effects, while reducing AA and, consequently, pro-inflammatory prostaglandins, leading to decreased neuroinflammation [23,24]. Thus, by simultaneously modulating central 2-AG levels and limiting AA formation, MAGL inhibition plays a key role in regulating neuroinflammation. This dual mechanism highlights its potential as a therapeutic strategy for neurodegenerative disorders where inflammation is a major contributing factor, such as Alzheimer’s disease (AD), Parkinson’s disease (PD) and multiple sclerosis (MS) [25,26,27]. In addition, MAGL blockade has also been shown to alleviate withdrawal symptoms, by increasing endocannabinoid levels, particularly those of 2-AG [15]. By elevating endocannabinoid levels, MAGL inhibitors enhance CB1-mediated signaling and significantly attenuate both behavioral and physiological symptoms of opioid withdrawal in preclinical models. Compared with direct cannabinoid receptor agonists, MAGL inhibitors may provide anti-withdrawal and antinociceptive effects with a lower incidence of cannabimimetic side effects, highlighting their potential as safer modulators of the endocannabinoid system in addiction therapy [28]. Moreover, endocannabinoid signaling appears to modulate dopaminergic pathways, which play a central role in reward processing and addicting behaviors. Inhibition of MAGL enhanced 2-AG-mediated CB1 receptor activation, thereby potentiating dopamine D1/D2 receptor-dependent neuronal firing in *nucleus accumbens* (NAc) neurons, and this supports the concept that the 2-AG/CB1 axis contributes to the regulation of reward-related neurocircuitry [29]. MAGL also has a central role in lipid metabolism by hydrolyzing monoacylglycerols (MAGs) into FFAs. In 2010, Nomura and colleagues reported high MAGL expression in aggressive human cancer cells and primary tumors [30], where the enzyme regulates a lipid signaling network that promotes migration, invasion and overall tumor growth [24,31]. Both pharmacological inhibition and genetic suppression of MAGL activity were shown to increase MAG levels while reducing FFAs levels in aggressive cancer cells. Notably, this reduction in FFAs was observed in malignant cells but not in normal tissues, where MAGL mainly regulates MAG levels [32]. These findings suggest that this lipid network is particularly important in aggressive cancer types. The inhibition of MAGL also leads to a significant decrease in several oncogenic lipids derived from FFAs, including lysophospholipids such as lysophosphatidic acid (LPA), lysophosphatidylethanolamine (LPE) and lysophosphatidylcholine (LPC), phosphatidic acid (PA) and prostaglandin E2 (PGE2) (Figure 2) [33]. Taken together, these findings position MAGL as a compelling and versatile therapeutic target across a wide spectrum of diseases.

### 1.3. Representative MAGL Inhibitors Reported Before 2020

Given MAGL central role in regulating 2-AG levels and its involvement in numerous physiological and pathological processes, considerable efforts have been focused on the development of potent and selective MAGL inhibitors to validate this enzyme as a therapeutic target. These studies led to the identification of several classes of compounds, generally classified according to their mechanism of action as irreversible or reversible inhibitors, each characterized by distinct pharmacological properties and specific biological implications [15,34]. This section briefly summarizes MAGL inhibitors reported up to 2020, highlighting their main structural and pharmacological features, providing the necessary background to contextualize the compounds developed in subsequent years.

#### 1.3.1. Irreversible Inhibitors

**Figure 3:**
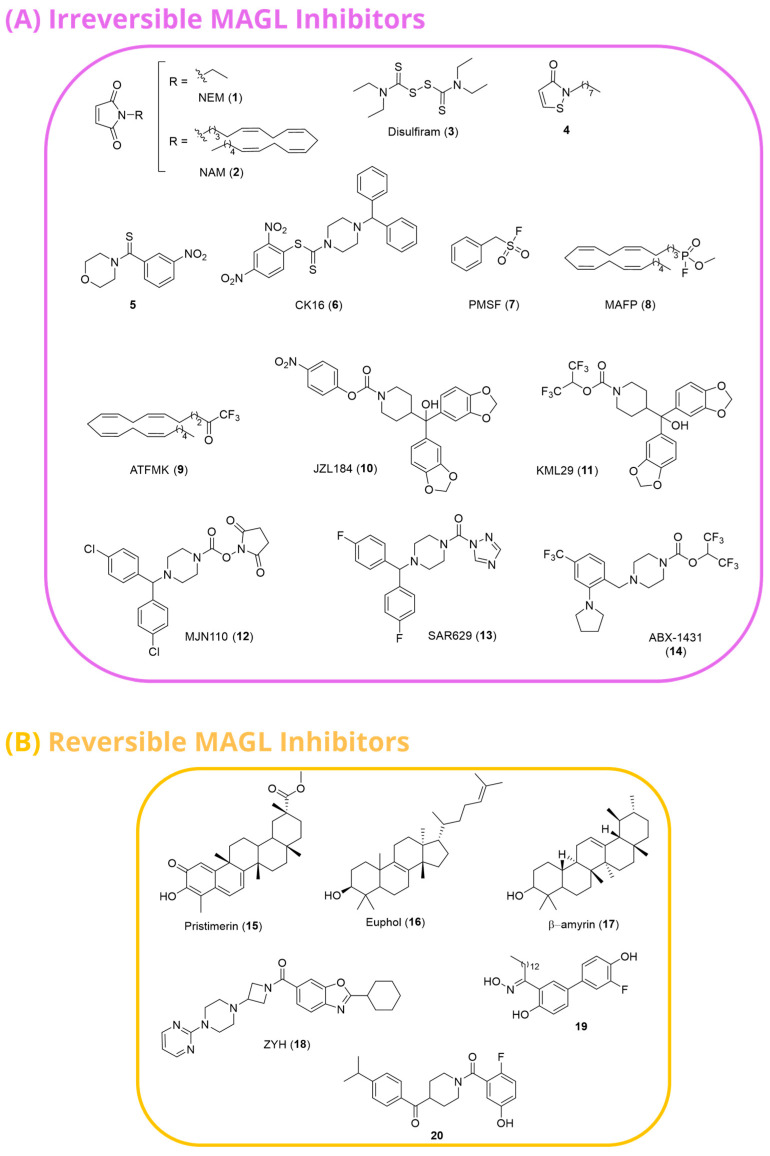
Structures of some representative irreversible (**A**) and reversible (**B**) MAGL inhibitors reported in the literature before 2020.

Irreversible inhibitors represent a widely studied class and are generally characterized by their ability to form stable covalent bonds with key residues of the enzyme, resulting in prolonged inhibition. Depending on the target residue, these compounds can be classified into cysteine-targeting inhibitors or inhibitors directed toward the catalytic serine [34,35]. Cysteine-targeting inhibitors covalently modify residues located near the catalytic site (notably Cys201, Cys208 and Cys242), affecting enzyme structure or stabilizing its inactive conformations. Among these, maleimide derivatives such as *N*-ethylmaleimide (NEM, compound **1**, Figure 3A) and *N*-arachidonoyl maleimide (NAM, compound **2**, Figure 3A) represent classical examples, as they react with the thiol group of Cys242 via Michael addition, forming *S*-alkylated adducts [12,35]. Similarly, disulfide-containing compounds, such as disulfiram (compound **3**, Figure 3A) and its analogues, inhibit MAGL through the formation of disulfide bonds with cysteine residues (Cys208 and Cys242) [36]. Additional scaffolds targeting sulfhydryl groups have also been developed, including isothiazolinone derivatives as compound **4** (Figure 3A) [37], which exhibit a partially reversible mechanism, aryl thioamide derivatives as compound **5** (Figure 3A) [38] and dithiocarbamates such as CK16 (**6**, Figure 3A) [39]. Another important subclass of irreversible inhibitors consists of compounds targeting the catalytic serine (Ser122), exploiting its nucleophilicity to form stable covalent adducts with electrophilic groups. Early examples included sulfonyl fluorides, fluorophosphonates and trifluoromethyl ketones represented by compounds **7**–**9**, respectively (Figure 3A) [40]. These compounds showed high potency but also significant non-specific reactivity toward other serine hydrolases, thereby limiting their selectivity. Subsequent development of new electrophilic scaffolds led to significant improvements in pharmacological profiles. In particular, carbamates represented a major breakthrough in this field: JZL184 (compound **10**, Figure 3A) is characterized by high potency, good brain permeability and marked selectivity over FAAH, acting through irreversible carbamoylation of Ser122 [41]. Further optimization led to derivatives such as KML29 (**11**, Figure 3A) [42] and MJN110 (**12**, Figure 3A) [43], which display excellent selectivity and in vivo activity. In parallel, urea-based inhibitors have also made important contributions, as exemplified by SAR629 (**13**, Figure 3A), which showed selective inhibitory activity against other enzymes of the ECS and whose crystal structure confirmed the formation of a covalent adduct with the catalytic serine [35,44]. Among more recent developments, ABX-1431 (**14**, Figure 3A) represents a significant advance due to its high selectivity, favorable pharmacokinetic properties and its evaluation in clinical studies for neurological disorders [45,46].

#### 1.3.2. Reversible Inhibitors

Despite their high efficacy, irreversible inhibition of MAGL may be associated with adverse effects related to prolonged activation of the endocannabinoid system, including CB1 receptor desensitization, as well as tolerance and dependence [47]. Prior to 2020, research efforts were primarily focused on the development of irreversible inhibitors, whereas reversible inhibitors received comparatively less attention and were underrepresented in the literature. Since 2020, however, increasing interest has been directed toward the development of reversible inhibitors, which enable transient modulation of enzymatic activity while preserving system homeostasis; these compounds will be discussed in detail in Section 2.2.

The pre-2020 scientific literature reports several classes of reversible inhibitors, each characterized by distinct advantages in terms of efficacy, selectivity and pharmacodynamic profile. Early examples of this class include natural terpenoids such as Pristimerin (compound **15**, Figure 3B) and Euphol (compound **16**, Figure 3B), which exhibit nanomolar inhibitory activity and interact with the hydrophobic pocket located within the enzyme “lid” domain, likely involving cysteine residues [48]. The triterpene β-amyrin (compound **17**, Figure 3B) was identified as a reversible inhibitor, although with lower micromolar potency [49]. In addition to natural compounds, several synthetic reversible inhibitors with different binding modes have been developed. Among these, piperazinyl-azetidinyl amides, including ZYH (compound **18**, Figure 3B), represent a notable example [50]. ZYH structural characterization enabled a high-resolution co-crystal structure with *h*MAGL, providing deeper insights into MAGL conformational flexibility, particularly within the catalytic site upon ligand engagement, and offered a valuable framework for the rational development of novel selective inhibitors [51]. Further advances in the development of reversible inhibitors have been achieved through the synthesis of several innovative scaffolds, including salicylketoxime derivatives (compound **19**, Figure 3B) [52] and benzoylpiperidine derivatives (**20**, Figure 3B) [53]. These compounds display low nanomolar inhibitory activity along with good selectivity over other enzymes of the ECS.

## 2. MAGL Modulators (2020–2026)

Considering the growing therapeutic relevance of modulating MAGL, significant efforts have been devoted in recent years to the development of novel strategies capable of targeting this key enzyme at the interface between the ECS and lipid metabolism. Advances in medicinal chemistry, together with the increasing application of innovative computational approaches and biochemical methods to assess MAGL activity [54], have enabled the identification of a wide range of selective MAGL modulators. These include not only classical enzyme inhibitors but also emerging strategies such as targeted protein degradation (TPD). It should be noted that, throughout this review, the term “selective MAGL modulators” refers broadly to compounds developed or investigated with the aim of modulating MAGL activity. While many of the reported molecules have demonstrated selectivity toward MAGL, others remain insufficiently characterized from a selectivity standpoint or have been shown to interact with additional biological targets.

In this section, we provide a comprehensive overview of selective MAGL modulators reported in the literature from 2020 to the present, excluding compounds described exclusively in patent literature and MAGL probes as this area has been recently reviewed elsewhere [55]. The modulators are first classified according to the type of interaction with the enzyme, distinguishing irreversible MAGL inhibitors (Section 2.1), reversible MAGL inhibitors (Section 2.2) and MAGL inhibitors with an undefined mechanism of action (Section 2.3). For consistency, compound numbering used in this review follows a sequential order independent of the original numbering reported in the source publications, except where specific compound identifiers are commonly used in the literature. Within each category, compounds are first distinguished according to their origin (natural compounds or synthetic molecules) and subsequently organized by chemical scaffold. Whenever possible, the compounds are discussed in chronological order, from the earliest reported examples to the most recent ones.

This classification allows a clear overview of the structural diversity and mechanistic strategies that have emerged in the development of selective MAGL modulators in recent years and could assist researchers in guiding the rational design and optimization of new MAGL-targeting agents with greater potential for successful therapeutic development.

### 2.1. Irreversible MAGL Inhibitors

Although irreversible MAGL inhibitors have been associated with undesired effects such as pharmacological tolerance, physical dependence and CB1 receptor desensitization [47] as discussed in the Introduction section, their development remains an active and relevant area of research. This sustained interest is largely driven by the exceptionally high inhibitory potency typically achieved by covalent inhibitors, often in the low-nanomolar or sub-nanomolar range. Irreversible inhibition therefore represents an effective strategy to ensure sustained target engagement and strong in vivo efficacy, even at low concentrations. As illustrated by several studies published between 2020 and 2026 and analyzed in the following sections, new irreversible MAGL inhibitors based on fully synthetic small molecule scaffolds have been reported, underscoring the continued relevance of this medicinal chemistry approach.

#### 2.1.1. Carbamates

**Figure 4:**
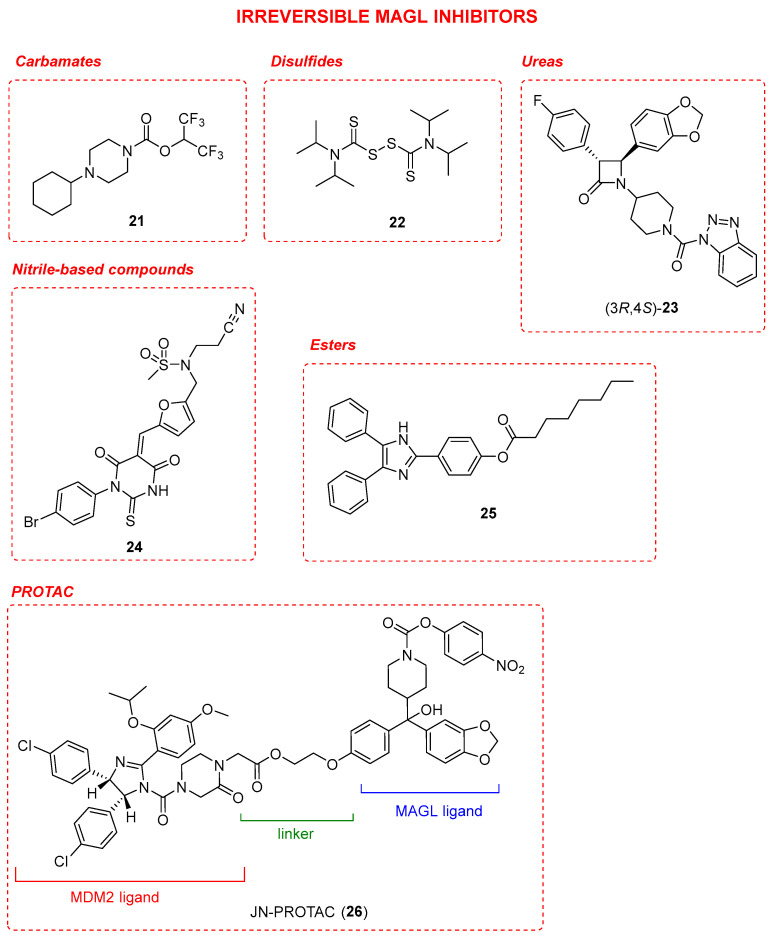
Irreversible MAGL inhibitors **21**–**25** and anti-MAGL PROTAC **26**.

In 2025, Hassen and co-workers reported an integrated de novo drug design strategy to identify novel MAGL inhibitors that are readily synthesizable using a limited in-house building block library with the aim of reducing costs, leading time and chemical waste in drug discovery process [56]. Following a multi-objective generative workflow combining a MAGL-targeted Quantitative Structure–Activity Relationship (QSAR) model with a computer-aided synthesis planning (CASP)-based synthesizability score, a small set of candidate molecules was prioritized for experimental validation. The selected compounds were synthesized according to CASP-suggested short synthetic pathways and subsequently evaluated for their MAGL inhibitory activity using a fluorescence-based assay with the natural substrate 2-AG in MAGL overexpressing membrane preparations. Glycerol production was quantified via glycerol kinase, glycerol-3-phosphate oxidase and horseradish peroxidase system using the fluorescent red dye Amplifu™ Red and IC_50_ values were determined from concentration-response curves. Among the three de novo designed and tested compounds, hexafluoroisopropyl carbamate **21** (Figure 4) displayed the most potent inhibitory activity, with an IC_50_ value of 1 µM. Although the inhibitory mechanism of compound **21** against MAGL was not investigated through specific mechanistic studies, its structural similarity to carbamate-based scaffolds, such as the well-known irreversible MAGL inhibitor ABX-1431 (compound **14**, Figure 3A) [45], suggests that it may behave as an irreversible MAGL inhibitor. Overall, this study underscores the value of integrating generative modeling with CASP to enable the efficient identification of synthetically accessible MAGL inhibitors, highlighting the relevance of computationally guided synthetic design strategies in drug discovery.

#### 2.1.2. Disulfides

In 2021, Omran and colleagues reported the design, synthesis and pharmacological evaluation of new disulfiram-derived thiuram disulfides as selective MAGL inhibitors [57] aiming to overcome the limited target selectivity of disulfiram (compound **3**, Figure 3A), a potent irreversible MAGL inhibitor that exerts its activity through the carbamylation of cysteine residues Cys208 and Cys242 near the catalytic site of the enzyme [58]. Notably, disulfiram displays low selectivity, as it also exhibits significant inhibitory activity against FAAH [36]. The structural optimization adopted in this study focused on the systematic replacement of the four *N*-ethyl substituents of disulfiram to modulate steric bulk, polarity and hydrophobicity. Among the synthesized derivatives, compound **22** (Figure 4)**,** in which the ethyl groups were replaced by *iso*-propyl substituents, showed an improved selectivity profile. In vitro enzymatic assays were performed using *h*MAGL and the compounds were compared to the reference irreversible MAGL inhibitor JZL184 (compound **10**, Figure 3A) [41] as the positive control. Selectivity was evaluated in parallel using recombinant human FAAH (*h*FAAH) in a fluorometric assay based on 7-amino-4-methylcoumarin (AMC)-arachidonoyl amide. Disulfiram displayed an IC_50_ value of 0.95 µM on MAGL, whereas compound **22** showed a slightly higher IC_50_ value of 1.89 µM, indicating a reduction in potency. Nevertheless, disulfiram inhibited FAAH with an IC_50_ of 36.20 µM, while compound **22** showed no detectable inhibition at the tested concentrations. This result demonstrated a clear increase in selectivity for MAGL over FAAH for compound **22**. The covalent and irreversible mechanism of inhibition was supported by reversibility experiments performed on a related analogue from the same series, which differs from **22** by the presence of an *iso*-butyl instead of an *iso*-propyl substituent. In the presence of the reducing agent 1,4-dithio-*D*,*L*-threitol (DTT), enzymatic activity was restored, consistent with a cysteine-targeting mechanism characteristic of the parent compound disulfiram and supporting an irreversible mode of inhibition of the disulfide analogue.

#### 2.1.3. Ureas

In 2024, Butini and co-workers reported a new class of irreversible MAGL inhibitors based on a diphenylazetidin-2-one scaffold, designed to mimic the characteristic “Y-shaped” arrangement of reference covalent inhibitors such as JZL184 (compound **10**, Figure 3A) [41] and second-generation triazole-ureas [35,44,59,60]. The design rationale relied on the use of a rigid *β*-lactam core capable of properly orienting two aryl substituents within the catalytic pocket of MAGL, while positioning a carbamoylating electrophilic center near the catalytic Ser122. Fine modulation of C3 and C4 substituents, together with variation in the leaving group (1,2,4-triazole, 1,2,3-triazole or 1,2,3-benzotriazole), enabled an extensive structure–activity relationship (SAR) analysis. Most compounds displayed nanomolar or sub-nanomolar inhibitory potency toward *h*MAGL and recombinant rat MAGL (*r*MAGL). Within this series, compound **23** (Figure 4) emerged as the most promising candidate. In its racemic form, (±)-**23** exhibited IC_50_ values of 12.19 and 0.22 nM against *h*MAGL and *r*MAGL, respectively. Enantiomeric separation revealed a marked stereoselective interaction: the eutomer (3*R*,4*S*)-**23** showed IC_50_ values of 0.021 and 0.240 nM against *h*MAGL and *r*MAGL, respectively, being approximately 900-fold more potent than its distomer on both human and murine forms of the enzyme. The eudysmic ratio was significantly higher than that observed for the previously developed reference compound of this study, highlighting a pronounced chiral cliff associated with the introduction of the benzotriazole leaving group. Time-dependent and dilution assays confirmed the irreversible mechanism of action, as enzymatic activity was not fully recovered 60 min after dilution. X-ray crystallographic structures of closely related analogues of the series in complex with *h*MAGL demonstrated covalent carbamylation of Ser122 and confirmed the enzyme preference for the (3*R*,4*S*) configuration. Concerning selectivity, (±)-**23** displayed weak affinity for CB1 and CB2 and a favorable profile in a panel of 50 off-targets. In competitive activity-based protein profiling (ABPP) experiments on 44 serine hydrolases in the murine brain proteome (±)-**23**, tested at concentrations ranging from 0.1 to 1 μM, mainly targeted MAGL, with limited interactions observed for ABHD6, ABHD12 and LYPLA2 (lysophospholipase II). In in vivo experiments, intraperitoneal administration of (±)-**23** (5 mg/kg) resulted in approximately 50% inhibition of brain MAGL activity after 1 h injection, accompanied by more than a 10-fold increase in 2-AG levels and a marked reduction in free AA, without significant alterations in other ethanolamides levels. Collectively, these results support diphenylazetidin-2-one derivatives as effective in vivo irreversible MAGL inhibitors and valuable tools for further pre-clinical investigation.

#### 2.1.4. Nitrile-Based Compounds

In 2025, Wang and colleagues discovered a novel class of irreversible MAGL inhibitors through a virtual screening approach based on the SCARdock protocol, a web free server for screening covalent ligands [61,62]. Among the 113 selected hits, five compounds were identified as promising MAGL inhibitors, with compound **24** (Figure 4) emerging as the most potent in biochemical assays. **24** inhibited MAGL in a concentration-dependent manner, displaying a IC_50_ value of 2.05 μM. Its modest inhibitory activity increased with longer pre-incubation times suggesting an irreversible inhibition, and kinetic analysis was consistent with a covalent mechanism of action. Time-dependence was confirmed using an orthogonal high performance liquid chromatography (HPLC)-based assay in which residual substrate was quantified after 0, 30 and 60 min pre-incubation. Compound **24** induced a progressive decrease in MAGL catalytic activity, in agreement with the spectrophotometric data. Functional irreversibility was further evaluated by a jump-dilution experiment as no recovery of enzymatic activity was observed after dilution. Target binding was examined by a gel-based thermal shift assay: upon incubation with **24** (600 μM), the melting temperature of MAGL increased, indicating enhanced thermal stability consistent with ligand binding. Notably, MALDI-TOF, a mass spectrometry technique that identifies biomolecules through laser ionization and mass-to-charge ratio measurement, did not reveal a detectable mass shift for compound **24**, suggesting either a reversible covalent interaction or limited stability of the covalent adduct, consistent with the moderate electrophilicity of the nitrile warhead. Docking analysis suggested that **24** binds within the MAGL active site through a network of hydrogen bonds and hydrophobic interactions. In particular, the oxygen atom and the nitrogen atom of the sulfonamide moiety form hydrogen bonds with His131 and Ala61, respectively, while additional hydrophobic contacts with Leu215, Leu223, Leu251, and Ile189 may contribute to stabilization of the ligand within the catalytic pocket. According to the mechanism proposed by the authors, the nitrile warhead undergoes nucleophilic attack by the catalytic Ser122 residue of MAGL, leading to the formation of a covalent enzyme-inhibitor adduct and consequent enzyme inactivation. However, the proposed mechanism still requires further clarification, since covalent adduct formation was experimentally confirmed only for one analogue of the series, whereas no detectable mass shifts were observed for **24** in the MALDI-TOF analyses, possibly due to the reversible nature or lower reactivity of the nitrile warhead. In cellular models, compound **24** demonstrated antiproliferative activity in triple-negative breast cancer MDA-MB-231 cells, with an EC_50_ value of 33.23 μM after 72 h of treatment. Moreover, LC-MS quantification of intracellular lipids showed a significant increase in 2-AG levels following 72 h exposure to compound **24** (50 μM), confirming effective inhibition of the MAGL pathway in a cellular context. These findings validated the SCARdock protocol as a powerful strategy for the discovery of MAGL inhibitors. However, the absence of a detectable mass shift in MALDI-TOF analyses underscored the need for further investigations to confirm **24**’s mechanism of inhibition.

#### 2.1.5. Esters

Alongside the discovery of selective MAGL inhibitors, substantial efforts have been devoted to the establishment and refinement of robust biochemical methodologies for the precise evaluation of MAGL enzymatic activity [54]. Accordingly, in 2025 Ottria and colleagues serendipitously discovered a novel class of MAGL inhibitors originating from an approach initially conceived for the development of chemiluminescent probes for MAGL assay [63]. The rationale of the study relied on the synthesis of lophine-based ester derivatives designed as chemiluminescence pro-enhancers within the luminol-H_2_O_2_-horseradish peroxidase (HRP) system, a sensitive enzymatic detection platform in which HRP catalyzes the oxidation of luminol by hydrogen peroxide to produce light, expected to release the active enhancer upon MAGL hydrolysis. However, the absence of a chemiluminescent signal led to a detailed investigation of the interaction between lophine-based ester derivatives and the enzyme and revealed a pronounced MAGL inhibitory activity. Among the developed lophine-based esters, compound **25** (Figure 4), corresponding to the octanoic acid ester of 2-(4-hydroxyphenyl)-4,5-diphenylimidazole, emerged as the most promising candidate. In fluorometric assays based on the hydrolysis of 7-hydroxyresorufinyl octanoate (7-HRO), compound **25** displayed a concentration-dependent inhibition of MAGL, with an IC_50_ value of 21 µM. The irreversible nature of its inhibition was demonstrated through dilution experiments, in which *h*MAGL pre-incubation with different concentrations of compound **25**, followed by a 15-fold dilution and a second inhibitor addition, resulted in no recovery of enzymatic activity. Enzyme kinetic analyses demonstrated a competitive inhibition mechanism, characterized by a 4-fold increase in *K*_m_ without significant changes in *V*_max_, as confirmed by Lineweaver-Burk plots. Molecular docking studies, performed with KDeep docking tools, supported experimental data, revealing a high predicted binding affinity for compound **25**, with a ΔG of −10.368 kcal/mol, predicted p*K*_d_ value of 7.6801 and a ligand efficiency (LE) value of −0.334 kcal/mol. It should be noted that these values were obtained from computational predictions and do not represent experimentally determined binding affinities. At the structural level, compound **25** establishes H-bond interactions through its ester moiety with Arg57 while the imidazole core interacts with the catalytic residue Ser122. In addition, a phenyl ring of the lophine scaffold is involved in a π-sigma interaction with Ala51. The aromatic scaffold is further stabilized by hydrophobic interactions with Leu148, Leu213, Leu205, Leu241 and Ile179. The octanoyl aliphatic chain extends into MAGL lipophilic channel, where it establishes additional hydrophobic interactions with Leu184, Tyr194, Lys273, supporting favorable accommodation of the ligand within the binding pocket. Taken together, these results identify lophine-based esters as a promising new class of irreversible MAGL inhibitors and pave the way for further structural optimization aimed at improving potency and pharmacological properties.

#### 2.1.6. Miscellaneous

Beyond classical small molecule inhibitors, recent years have witnessed the emergence of innovative chemical approaches aimed at regulating MAGL function through alternative mechanisms. In particular, targeted protein degradation (TPD) approaches, such as PROteolysis TArgeting Chimeras (PROTACs) [64], have broadened the range of pharmacological strategies available to study and modulate MAGL activity. PROTAC development consist of an emerging therapeutic approach that induces selective protein degradation via the ubiquitin-proteasome system (UPS). PROTACs are bifunctional molecules that simultaneously engage a protein of interest and an E3 ubiquitin ligase, promoting the formation of a ternary complex that facilitates ubiquitination and subsequent proteasomal degradation of the target protein [65].

In this context, very recently, Yuan and colleagues reported a rationally designed PROTAC capable of inducing targeted degradation of MAGL in the context of glioblastoma (GBM), the most aggressive and lethal form of primary brain cancer in adults [65]. GBM is characterized by pronounced molecular heterogeneity and the presence of glioblastoma stem cells (GSCs), which play a critical role in tumor initiation, progression and therapeutic resistance [66]. In this study, MAGL was selected as a therapeutic target due to its elevated expression in GSCs and its pivotal role in regulating GSC self-renewal and tumorigenicity [67]. The authors developed JN-PROTAC (**26**) (Figure 4), generated through a computer-aided rational design approach, representing the first anti-MAGL PROTAC ever reported in the literature. The MAGL-targeting warhead was derived from a structurally modified analog of JZL184 (compound **10**, Figure 3A) [41] in which non-essential moieties were adapted to allow linker conjugation while preserving the chemical features required for MAGL binding. For E3 ligase recruitment, the E3 ubiquitin-protein ligase mouse double minute 2 homolog (MDM2) was selected based on bioinformatic analyses of The Cancer Genome Atlas (TCGA) and the Clinical Proteomic Tumor Analysis Consortium (CPTAC) databases, demonstrating its high expression in GBM relative to normal tissues. A Nutlin-3-derived MDM2 ligand was conjugated to the MAGL warhead through an appropriate linker, yielding a bifunctional molecule capable of simultaneously promoting MAGL degradation and indirectly enhancing p53 signaling via functional engagement of MDM2. Cellular assays demonstrated that JN-PROTAC (**26**) efficiently induced MAGL degradation in GSCs in a dose-dependent manner. Quantitative analysis of immunoblot data revealed a DC_50_ of 60.28 nM in GSC 528 cells and 373.6 nM in GSC X01 cells, with maximal degradation (*D*_max_) reaching approximately 95% and 92.95%, respectively. MAGL degradation occurred rapidly, becoming evident within 8 h of treatment, and was dependent on the ubiquitin-proteasome pathway, as confirmed by rescue experiments using the inhibitor of the ubiquitin-proteasome system TAK-243 and the proteasome inhibitor MG-132. Functionally, JN-PROTAC (**26**) markedly suppressed GSC proliferation, significantly impaired sphere-forming capacity and induced apoptosis in a dose-dependent manner. Furthermore, in both sub-cutaneous and orthotopic patient-derived xenograft GBM models, JN-PROTAC (**26**) treatment resulted in significant tumor growth inhibition and prolonged survival, accompanied by efficient MAGL degradation and activation of apoptotic pathways, highlighting the therapeutic potential of MAGL-directed PROTACs in GBM. Overall, this study provides the first evidence that MAGL can be effectively targeted for proteasomal degradation via a PROTAC approach, establishing a foundation for further development of MAGL-directed degraders and expanding the potential of TPD strategies in this field.

### 2.2. Reversible MAGL Inhibitors

Considering the limitations and adverse effects associated with the chronic blockade of MAGL produced by irreversible inhibitors, the development of reversible MAGL inhibitors has received growing attention in recent years. Unlike covalent inhibitors, reversible compounds allow a more controlled modulation of enzymatic activity, potentially preserving the physiological balance of the endocannabinoid system while still achieving significant pharmacological effects. Consequently, considerable efforts have been devoted to the identification of reversible MAGL inhibitors displaying high potency and improved safety profiles. As discussed in the following sections, this class encompasses a large and structurally diverse set of molecules, including both natural compounds and, more prominently, synthetic small molecules. Among the latter, piperidine- and piperazine-based scaffolds have emerged as some of the most widely explored chemotypes.

#### 2.2.1. Natural Compounds

##### Flavones

**Figure 5:**
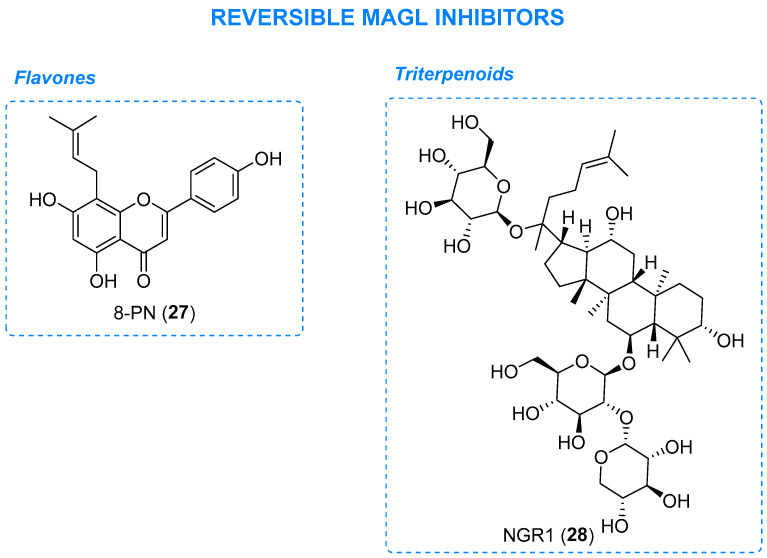
Natural reversible MAGL inhibitors.

In 2021, Tung and colleagues identified a natural and reversible MAGL inhibitor using a structure-based computer-aided drug design approach. The study exploited the X-ray crystal structure of the *h*MAGL-E3A complex (PDB ID: 6BQ0) [68], where E3A acts as a covalent inhibitor, for the generation of a receptor-ligand pharmacophore model (Phar-MAGL), constructed through the analysis of non-covalent interactions between the enzyme and the co-crystallized inhibitor [69]. The Phar-MAGL model defined the essential pharmacophoric features as one hydrogen bond acceptor and three hydrophobic regions located within the catalytic pocket of *h*MAGL and was employed as a primary filter for the virtual screening of 68,285 natural products. Pharmacophore-based screening results were integrated with high-throughput molecular docking (LibDock), leading to a selection of candidates based on pharmacophore matching, docking scores, and structural criteria. Subsequent biochemical validation identified compound **27** (Figure 5) as the most active compound, exhibiting dose-dependent inhibition of *h*MAGL with an IC_50_ value of 9.5 μM. Compound **27** was identified as 8-prenylnaringenin (8-PN), a prenylated flavonoid isolated from *Humulus lupulus* L. Molecular dynamics simulations followed by computational studies revealed that 8-PN (**27**) establishes a well-defined interaction pattern within MAGL active site. In particular, the phenolic hydroxyl group located on the terminal phenyl ring forms an H-bond with the catalytic residue Ser122, while the carbonyl group forms an H-bond with Tyr194. Moreover, the two hydroxyl substituents on the fused aromatic core participate in additional H-bond interactions with Ile179, Leu176 and Gly177. Further stabilization is provided by hydrophobic contacts involving the aromatic scaffold and the side aliphatic chain with Ile179, Leu205, Leu213, and Leu241.

As a phytoestrogen displaying high affinity for the estrogen receptor α, 8-PN (**27**) has been associated with neuroprotective effects related to estradiol signaling [70]. In the present study, 8-PN (**27**) was also found to inhibit *h*MAGL activity, and since both estrogen-related neuroprotective effects and MAGL inhibition have been associated with reduced neuroinflammation and neurodegenerative processes, these findings further support the potential therapeutic role of 8-PN (**27**) in Alzheimer’s disease.

##### Triterpenoids

Paclitaxel is a widely used chemotherapeutic agent that frequently causes chemotherapy induced peripheral neuropathy (CIPN), characterized in particular by paclitaxel-induced mechanical allodynia (PIMA) [71]. An increasing number of studies claim that dysregulation of the endocannabinoid system is implicated in CIPN pathogenesis [72]. Paclitaxel increases peripherical MAGL activity, which accelerates the degradation of 2-AG, thereby reducing endocannabinoid receptor (CB1 and CB2) signaling and enhancing pain sensitivity [71]. Given that certain plant derived triterpenoids, such as pristimerin (compound **15**, Figure 3B) [48], inhibit MAGL and attenuate neuropathic pain [73], in 2025 Al-Musailem and collaborators hypothesized that Notoginsenoside R1 (NGR1, compound **28**, Figure 5), a triterpenoid metabolite extracted from *Panax notoginseng*, may have antiallodynic effects through similar mechanisms [74]. The authors investigated whether the prophylactic administration of NGR1 (**28**) could prevent the development of PIMA in a murine model and whether this effect was associated with modulation of MAGL activity. The ability of NGR1 (**28**) to reduce mechanical allodynia was evaluated by assessing nociceptive responsiveness using validated behavioral tests that quantify paw withdrawal thresholds in response to gradually increasing mechanical *stimuli*. Paclitaxel administration induced a significant reduction in withdrawal thresholds, confirming the development of mechanical allodynia and validating the experimental model. Prophylactic treatment with NGR1 (**28**) markedly prevented this decrease in a dose-dependent manner, restoring withdrawal thresholds to values comparable to those observed in vehicle-treated control animals. Importantly, NGR1 (**28**) administered in the absence of paclitaxel did not affect baseline mechanical thresholds, indicating that its protective effects cannot be explained by non-specific analgesia. Instead, these data support a context-dependent effect in which NGR1 (**28**) selectively counteracts pathological mechanisms underlying paclitaxel-induced neuropathic pain. To investigate the underlying mechanisms, the authors examined the interaction between NGR1 (**28**) and MAGL by performing in silico docking studies based on the X-ray crystal structure available in the Protein Data Bank (PDB: 5ZUN) as the starting model [75]. This analysis revealed that NGR1 (**28**) had a lower affinity for MAGL compared to pristimerin (**15**). However, NGR1 (**28**) interacted with the same residues as pristimerin, as well as with additional aminoacids not involved in pristimerin binding. Complementary in vitro assays using *h*MAGL confirmed that NGR1 (**28**) inhibited the enzyme activity in a reversible and time-dependent manner, with a lower inhibition at longer incubation times. The activity of *h*MAGL was measured at 2 and 30 min after incubation with NGR1 (**28**): after 30 min the inhibitory effect was markedly reduced, suggesting that the enzyme activity was not permanently blocked and could recover over time. The concentration-response relationship was non-linear, with lower concentrations having a greater inhibitory effect than higher concentrations, a pattern suggesting allosteric modulation rather than classical competitive inhibition. Notably, no IC_50_ value on the isolated enzyme was reported for NGR1 (**28**) in the original study: this is consistent with the atypical concentration-response relationship observed, which does not permit conventional sigmoidal curve fitting, thus preventing reliable IC_50_ estimation.

Despite these findings, ex vivo analysis of peripheral tissue showed that NGR1 (**28**) only partially attenuated paclitaxel-induced increases in MAGL activity, suggesting that MAGL modulation alone does not fully explain the observed behavioral effects. The authors proposed that NGR1 (**28**) exerts its antiallodynic effects through additional neuroprotective mechanisms. Indeed, as shown in prior literature, NGR1 (**28**) has been shown to reduce oxidative stress, suppress neuroinflammatory signaling and inhibit neuronal apoptosis processes that play central roles in paclitaxel-induced peripheral nerve damage [76,77,78].

Taken together, these findings indicate that NGR1 (**28**) prevents PIMA through a multimodal mechanism involving partial modulation of the endocannabinoid system, by inhibiting MAGL, and broader antioxidant and anti-inflammatory effects.

#### 2.2.2. Synthetic Compounds

##### Diazetidinyl Diamides

**Figure 6:**
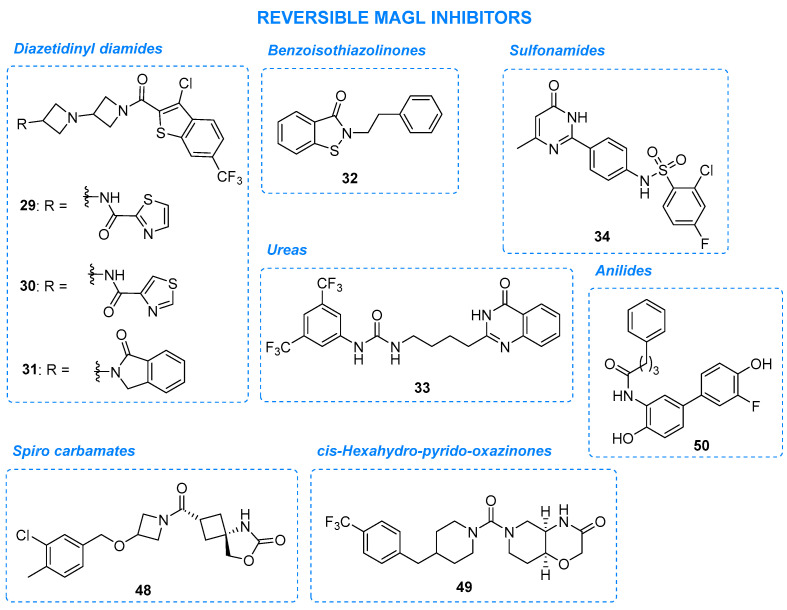
Synthetic reversible MAGL inhibitors, part 1.

In 2020, Zhu and co-workers reported the development of a novel class of diazetidinyl diamides as reversible MAGL inhibitors [79], achieved through the optimization of a potent reversible scaffold previously reported by the same research group [51]. The first series of compounds, characterized by a linear diazetidinyl diamide scaffold, showed a clear SAR, with thiazolyl substitution leading to a marked increase in potency over the corresponding analogues in which the thiazole was replaced by a phenyl ring. Within this series, compounds **29** and **30** (Figure 6) emerged as the most active inhibitors, displaying IC_50_ values below 5 nM on *h*MAGL, together with high functional efficacy in the rat brain 2-AG accumulation assay when tested at 1 μM concentration (460% and 477% for compounds **29** and **30**, respectively). In contrast, the corresponding phenyl analogues showed substantially lower activity. Moreover, the authors developed a second series of cyclic diazetidinyl amides. In this series, compound **31** (Figure 6) emerged as the most potent inhibitor, with biological activity comparable to that of the previously reported derivatives **29** and **30**, exhibiting an IC_50_ value below 5 nM in the enzymatic assay and inducing a marked increase in brain 2-AG levels at 1 μM concentration (449%). X-ray crystallographic studies confirmed a non-covalent binding mode within the MAGL catalytic pocket. The azetidine-amide carbonyl is oriented toward the oxyanion hole and forms an H-bond with the backbone NH group of Met123, adjacent to the catalytic residue Ser122. Additionally, the thiazole-amide carbonyl establishes an H-bond with the guanidine group of Arg57, while π–π stacking interactions are observed with Tyr194. All the selected compounds displayed high selectivity for MAGL over FAAH (IC_50_ > 10 μM) and good blood–brain barrier (BBB) penetration following oral administration in rats (10 mg/kg). Collectively, these results highlight the potential of diazetidinyl diamides-based scaffolds as effective reversible MAGL inhibitors with suitable pharmacokinetic properties.

##### Benzoisothiazolinones

In 2020, Castelli and collaborators, discovered benzisothiazolinone (BTZ) derivatives as potent MAGL inhibitors [80]. This work started from prior evidence showing that MAGL activity is regulated by cysteine residues located outside the catalytic site, in particular Cys201 and Cys208, which undergo reversible sulfenylation under oxidative conditions. This modification reduces enzymatic activity by interfering with substrate recruitment [81]. The authors hypothesized that small molecules selectively targeting these regulatory cysteines could act as reversible allosteric MAGL inhibitors, mimicking endogenous redox regulation. Focusing on the BTZ scaffold, previously shown to interact with regulatory cysteines [82], they conducted a comprehensive SAR study by systematically modifying substituents on both the nitrogen atom and the benzene ring of the BTZ core. The synthesized compounds were evaluated for their ability to inhibit *r*MAGL in vitro. Among the tested compounds, the *N*-phenethyl BTZ derivative, compound **32** (Figure 6) emerged as a particularly potent inhibitor (IC_50_ = 34.1 nM) and was selected for further mechanistic investigation. Site-directed mutagenesis of MAGL, in which Cys201, Cys208, or both residues were replaced by alanine, revealed a marked reduction in inhibitory potency, indicating that both cysteines contributed to its mechanism of action. Mass spectrometry analysis confirmed that compound **32** forms a reversible covalent adduct with Cys201, while other cysteine residues remained unmodified. Reactivity studies with glutathione further supported the ability of BTZ derivatives to undergo reversible thiol-based reactions. Molecular modeling and metadynamics simulations showed that the modification of Cys201 by BTZ derivatives stabilizes a closed conformation of the enzyme lid domain, limiting substrate recruitment from the membrane and reducing enzymatic activity. The biological relevance of this mechanism was examined in vitro and in vivo models. In neuronal cells exposed to oxidative stress, compound **32** reduced cell damage, while systemic administration in mice led to increased brain levels of 2-AG, despite limited brain penetration, indicating effective MAGL inhibition in vivo. Selectivity studies revealed that compound **32** did not significantly inhibit other key members of the ECS, including CB1 and CB2, DGL-*α* (diacylglycerol lipase-alpha), ABHD6 and FAAH, at concentrations effective for MAGL inhibition. Compound **32** showed negligible activity on cysteine-rich ion channels such as TRPA1 (transient receptor potential cation channel subfamily A member 1) and TRPV1 (transient receptor potential cation channel subfamily V member 1), indicating its selectivity on MAGL despite its thiol-reactive properties. Overall, these findings show that BTZ derivatives are potent, reversible and selective MAGL inhibitors that target the regulatory cysteines Cys201 and Cys208; moreover, they highlight the BTZ-based scaffold as a promising starting point for the development of redox-inspired MAGL inhibitors.

##### Ureas

In 2020, Dato and collaborators identified a new class of reversible MAGL inhibitors through a structure-based virtual screening strategy based on molecular docking studies using a MAGL co-crystal structure with a known reversible inhibitor, ZYH (compound **18**, Figure 3B) (PDB: 3PE6) [51,83]. Based on the in silico results, the authors synthesized 45 compounds belonging to eight different chemical classes: sulfonacetamides, oxalamides, squaramides, thiazoles, quinazolinones, imidazolinones, triazoles and naphthalenes. These compounds were tested for their inhibitory activity against *h*MAGL and, to assess selectivity, against *h*FAAH and murine cholesterol esterase (*m*CEase). This preliminary screening led to the identification of several compounds displaying good inhibitory activity toward MAGL. To confirm that this inhibition did not arise from non-specific aggregation, they also performed the experiments in the presence of the detergent Triton X-100, which resulted in a strong loss of inhibitory activity. Only three closely related compounds, belonging to the ω-quinazolinonylalkyl aryl urea class, retained their inhibitory activity toward *h*MAGL in the presence of the detergent. Structurally, these compounds differed only in the length of the alkyl linker connecting the quinazolinone core to the urea moiety. Among them, compound **33** (Figure 6) was identified as the most potent MAGL inhibitor with an IC_50_ value of 19.6 µM. For this reason, compound **33** was subjected to a more detailed kinetic analysis which showed that its inhibitory potency was independent of pre-incubation time, providing strong evidence of a reversible mode of inhibition. Molecular docking experiments further supported these findings by showing that the compound occupies the substrate-binding pocket of *h*MAGL in a binding mode comparable to that of known reversible inhibitor ZYH (compound **18**, Figure 3B) [50]. However, unlike ZYH, which establishes two hydrogen bonds with the oxyanion hole residues Ala51 and Met123, compound **33** lacks these interactions relying predominantly on lipophilic interactions. In particular, the quinazoline ring of compound **33** forms π-π stacking interactions with Tyr19, contributing to stabilization within the catalytic site. In conclusion, the authors demonstrated that the ω-quinazolinonylalkyl aryl urea moiety represents a promising lead structure for future optimization and biological investigations as MAGL inhibitors.

##### Sulfonamides

In 2020, Jha and co-workers identified a novel MAGL inhibitor through a pharmacophore-guided virtual screening strategy aimed at capturing the key interaction requirements of the enzyme catalytic site [84]. In particular, the receptor-based pharmacophore model was built by mapping the most relevant polar and hydrophobic hotspots within the binding pocket, highlighting hydrogen bond-donor and acceptor regions close to the oxyanion hole, together with hydrophobic features extending along the lipophilic channel of MAGL. These pharmacophoric elements were subsequently validated by docking and molecular dynamics simulations and then used to screen a commercial compound database, followed by further docking-based prioritization of the most promising hits. Among the selected candidates, compound **34** (Figure 6) emerged as the most active derivative and was experimentally confirmed as a MAGL inhibitor, displaying an IC_50_ value of 34.7 µM. Structurally, **34** is characterized by an *ortho*-chloro,*para*-fluoro-benzenesulfonamide moiety linked, through a phenyl spacer attached to the sulfonamide nitrogen, to a 6-methylpyrimidin-4(*3H*)-one ring. Kinetic studies and dilution experiments demonstrated a reversible and competitive mode of inhibition, as enzyme activity was fully restored after dilution of the enzyme-inhibitor complex, thus excluding an irreversible binding mechanism. Moreover, the inhibitory activity of **34** was not affected under reducing conditions (in presence of the thiol-containing agent DTT), indicating that the compound does not rely on interactions with cysteine residues for enzyme inhibition. Molecular modeling analyses were consistent with the pharmacophore-based predictions, showing that the sulfonamide moiety of **34** engages the polar region of the catalytic cavity through hydrogen-bond interactions while simultaneously occupying the hydrophobic channel with its aromatic moiety. This proposed binding mode stabilizes the ligand within the active site and supports its competitive inhibitory behavior. Overall, the identification of **34** highlights the ability of pharmacophore-guided virtual screening approaches to capture essential binding features of MAGL and to deliver chemically novel hit compounds suitable for further optimization.

##### Piperidines and Piperazines

**Figure 7:**
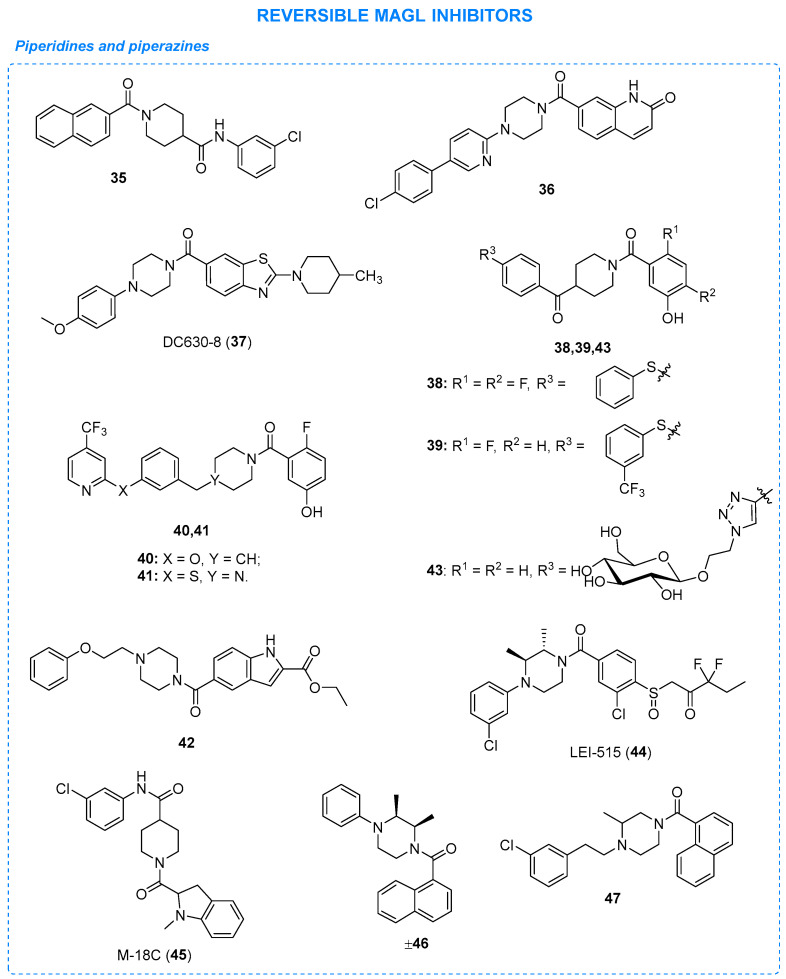
Synthetic reversible MAGL inhibitors, part 2.

In 2020, Zhi and co-workers reported the rational development of a novel class of reversible MAGL inhibitors [85], starting from well-known irreversible MAGL inhibitors MJN110 (compound **12**, Figure 3A) [43] and SAR629 (compound **13**, Figure 3A) [35,44]. The authors explored a first series of derivatives obtained through targeted scaffold modifications aimed at reducing covalent reactivity toward the catalytic Ser122 residue; however, this series generally exhibited limited inhibitory activity but provided preliminary structural insights to guide subsequent optimization. Based on molecular docking analyses and a refined SAR investigation, a second series of aryl formyl piperidine derivatives was subsequently designed, characterized by increased conformational rigidity and the presence of a dual amide system capable of optimal interactions within the MAGL oxyanion hole. This optimization pathway led to the identification of compound **35** (Figure 7), in which with the presence of a naphthalene moiety enabled an efficient occupation of the enzyme’s hydrophobic cavity. Compound **35** emerged as the most promising candidate, displaying potent inhibitory activity on *h*MAGL (IC_50_ = 14.75 nM) and high selectivity over FAAH (6780-fold) as well as cannabinoid receptors CB1 and CB2 (EC_50_ > 10 μM for both receptors). Reversibility was confirmed by dilution and pre-incubation experiments, while kinetic analyses demonstrated a competitive mode of inhibition, with a *K_i_* value of 13.4 nM and a α value greater than 10,000. Molecular docking studies elucidated the binding mode of compound **35**, highlighting the formation of two hydrogen bonds between the amide carbonyl group linked to the naphthalene ring and the backbones of Ala51 and Met123. Notably, the *m*-chloro-substituted aniline moiety occupied a closed hydrophobic pocket of the active site, establishing lipophilic interactions with Val191, Tyr194, Val270, and Lys273, as well as a π-π interaction with Tyr194. The naphthalene moiety occupies the larger hydrophobic cavity, establishing lipophilic interactions with Leu148, Leu213, and Leu241. From a pharmacological perspective, compound **35** exhibited a favorable profile, including good stability in human liver microsomes (88.46% remaining after 60 min of incubation), low cytotoxicity in murine fibroblasts (L929), and suitable in vivo pharmacokinetic properties. Following intragastric administration in rats (10 mg/kg), compound **35** reached a plasma *C*_max_ of 83.122 μg/L with a *T*_max_ of 34 min, a plasma half-life of approximately 5 h, and an oral bioavailability of 21.6%. Importantly, compound **35** demonstrated efficient BBB penetration, with a brain *T*_max_ of approximately 40 min, a brain half-life of about 2.5 h, and a relative brain bioavailability of 36.1%, higher than in plasma. Notably, compound **35** also exhibited significant antidepressant effects in a reserpine-induced murine model of depression, significantly reducing immobility time in both the forced swim and tail suspension tests following intragastric administration (5 mg/kg), with efficacy comparable to the positive control fluoxetine hydrochloride capsules.

Subsequently to this study, in 2022, Liu and co-workers further investigated a selected set of four reversible MAGL inhibitors among the series of the compounds previously reported by Zhi and colleagues in 2020 [85], using an integrated computational approach combining molecular docking, molecular dynamics simulations and binding free energy calculations based on the molecular mechanics/Poisson–Boltzmann surface area (MM/PBSA) method [86]. The rationale of this study was to elucidate how variations in the aryl formyl piperidine scaffold could affect binding affinity and inhibitor-enzyme complex stability. Among the four compounds analyzed, compound **35** (Figure 7) exhibited the most favorable binding profile, in line with the results previously reported by Zhi and colleagues. In particular, compound **35** established hydrogen bonds between the carbonyl oxygen of the naphthalene amide moiety and key residues as Ala51 and Met123 of the oxyanion hole, as well as a direct interaction with the catalytic residue Ser122. Collectively, these results defined the structural determinants contributing to the enhanced inhibitory profile of compound **35** and clarified the molecular basis of reversible MAGL inhibition within the piperidine-based framework. The combined kinetic, pharmacological and computational data supported a coherent mechanistic interpretation for this potent inhibitor.

In 2024, Hao and collaborators reported the discovery a novel class of reversible MAGL inhibitors, belonging to the (piperazine-1-carbonyl)-quinolin-2(1*H*)-one chemical class, with the aim of developing new antidepressant candidates [87]. Starting from the previously reported MAGL inhibitor **35** (Figure 7) [85], which showed good in vivo efficacy but poor metabolic stability, the authors applied a structure-based design and molecular hybridization strategy. Analyzing MAGL crystal structures, they identified key binding requirements, including a central carbonyl group to interact with the oxyanion hole and appropriately sized aromatic moieties to occupy the lipophilic tunnel and the hydrophobic pocket of the enzyme. To improve potency, they introduced a quinolin-2(1*H*)-one scaffold, inspired by the antidepressant brexpiprazole, a second-generation atypical antipsychotic drug approved for the treatment of major depressive disorder and schizophrenia [88] and linked it to a piperazine-based amide moiety. SAR studies revealed that electron-withdrawing and bulky substituents on the terminal phenyl ring, particularly at the *para* position, were crucial for strong activity. Through this optimization, the *para*-chloro substituted compound **36** (Figure 7) emerged as the most active compound, showing an IC_50_ value of 10.3 nM and high selectivity over FAAH and cannabinoid receptors. Compound **36** was subjected to reversibility analysis in dilution and in pre-incubation assays, and both assays confirmed the reversibility of the inhibition mechanism. Compound **36** also showed high metabolic stability, good brain penetration and overall, a very high oral bioavailability. These properties translated into a robust in vivo performance, with compound **36** increasing brain 2-AG levels and producing antidepressant effects without addictive behavior in animal models. Collectively, the authors demonstrated that compound **36** represents a great candidate for antidepressant therapy, supporting the (piperazine-1-carbonyl)-quinoline-2(1*H*)-one scaffold as a promising moiety for developing MAGL reversible inhibitors.

In 2021, Xiong and colleagues reported the discovery of a novel series of reversible MAGL inhibitors identified through structure-based virtual screening combined with biochemical analysis [89]. The virtual screening workflow involved the application of Lipinski’s rule of five and pan-assay interference compounds (PAINS) filtering to eliminate compounds with poor drug-likeness or known interfering structural alerts, followed by Glide SP docking of the compound library into the MAGL active site. The top 10,000 ranked compounds were clustered into 100 groups, and candidates with unfavorable shapes were excluded, resulting in the selection of 88 compounds for biological evaluation. Enzymatic inhibition assays conducted at 50 μM identified 15 compounds exhibiting inhibition rates greater than 60%, leading to the identification of a hit compound with an IC_50_ value of 5 μM. To further investigate the SAR, a series of analogues was generated through 2D similarity searching and then evaluated in enzymatic assays. Among these derivatives, DC630-8 (**37**, Figure 7) emerged as the most potent compound, displaying an IC_50_ value of 2.84 μM. The direct binding of DC630-8 (**37**) to MAGL was confirmed by surface plasmon resonance (SPR), yielding an equilibrium dissociation constant (*K*_d_) value of 0.71 μM. Molecular docking studies suggested that DC630-8 (**37**) occupies the catalytic pocket of MAGL, where the amide carbonyl group forms hydrogen bonds with residues Ala51 and Ser122 while the aromatic ring engages in π-π stacking interactions with Tyr194. In addition, the benzyl moiety is embedded within the hydrophobic cavity, where it establishes hydrophobic interactions with Glu53, Arg57, His121, Ile179, Ser181, Leu184, Glu190, Val191, Tyr194, Val270 and Lys273. The reversible mechanism of inhibition was supported by DTT and pre-incubation assays. Furthermore, DC630-8 (**37**) significantly increased intracellular 2-AG levels and dose-dependently suppressed the expression of pro-inflammatory cytokines in lipopolysaccharide (LPS) stimulated RAW264.7 macrophages, supporting its functional activity at the cellular level. Overall, this study provides a solid foundation for the optimization of novel reversible MAGL inhibitors and offers valuable insight into the anti-inflammatory potential associated with MAGL blockade.

In 2021, Granchi and collaborators expanded and optimized the previously developed class of benzoylpiperidine-based MAGL inhibitors, originally reported in 2019 [53]. This effort led to a second-generation class of benzoylpiperidine-based MAGL inhibitors and to the identification of compound **38** (Figure 7) as the most potent derivative of the series [90]. Compound **38** is a diphenylsulfide-benzoylpiperidine derivative bearing two fluorine atoms on the phenolic ring, positioned at the *ortho* and *para* positions relative to the phenolic hydroxyl group. In enzymatic assays, **38** displayed a strong inhibitory activity against *h*MAGL, with an IC_50_ value of 18 nM. Further biochemical investigations demonstrated that **38** behaves as a reversible and competitive MAGL inhibitor, as confirmed by pre-incubation, dilution and kinetic experiments. Molecular modeling and molecular dynamics simulations provided a structural rationale for this profile, showing that the phenolic group of **38** establishes a key hydrogen-bond network within the small polar cavity of the MAGL catalytic site through interactions with His272 and Glu53. In parallel, the central carbonyl group engages the oxyanion hole residues Ala51 and Met123 via stable hydrogen bonds, while the diphenyl-sulfide moiety deeply occupies the lipophilic channel at the entrance of the binding pocket, forming extensive hydrophobic interactions with residues such as Ala151, Phe159, Leu205, Leu213 and Leu241, that stabilize the ligand within the enzyme cavity. Selectivity toward the serine hydrolase family was further evaluated by ABPP, which confirmed that **38** selectively inhibits MAGL with negligible off-target involvement of other serine hydrolases. The inhibitory activity was also confirmed in intact cells, where **38** efficiently reduced MAGL activity in human monocytic cell line U937 cells, indicating effective cellular permeability and intracellular target engagement. Beyond its enzymatic profile, compound **38** displayed significant antiproliferative effects in different cancer cell lines, including human breast MDA-MB-231, colorectal HCT116, ovarian CAOV3 and SKOV3 cancer cells at micromolar concentrations (IC_50_ values ranging from 11 to 63 µM). In addition, **38** exerted a remarkable antiproliferative activity against pancreatic ductal adenocarcinoma primary cell cultures (PDAC3) characterized by high MAGL expression (IC_50_ = 14.6 µM). Notably, growth inhibition was also confirmed in more advanced biological system, such as patient-derived cancer organoids, further supporting the translational relevance of this scaffold.

In 2021, Bononi and co-workers optimized the previously identified diphenylsulfide-benzoylpiperidine scaffold [91] and among the newly synthesized derivatives, the *meta*-trifluoromethyl analogue **39** (Figure 7) emerged as the most potent compound, exhibiting a notable inhibitory activity with an IC_50_ value of 1.26 nM. Kinetic studies confirmed that **39** acts as a competitive and fully reversible inhibitor. Molecular modeling provided a structural rationale for its potency, showing that *meta*-substituted distal phenyl ring of the diarylsulfide portion is efficiently accommodated within a hydrophobic sub-pocket at the entrance of the catalytic cleft, forming extensive van der Waals interactions with amino acidic residues such as Leu148, Phe159, Leu213 and Val217. In parallel, the phenolic hydroxyl group contributes to the anchoring of the molecule by engaging the conserved hydrogen-bond network involving Glu53 and His272. Compound **39** displayed an excellent enzymatic selectivity profile, showing negligible inhibition of FAAH even at high micromolar concentrations. Beyond its biochemical potency, **39** also demonstrated antiproliferative effects in several cancer cell lines, with submicromolar activity in OVCAR5 ovarian cancer cells (IC_50_ = 0.32 µM), supporting the potential relevance of this scaffold in oncological contexts where MAGL plays a pathogenic role. In conclusion, compound **39** represented a highly optimized reversible MAGL inhibitor combining remarkable potency, selectivity and preliminary antiproliferative effects, thus consolidating the diphenylsulfide-benzoylpiperidine framework as a valuable starting point for further development.

One year later, the same research group developed a new series of benzylpiperidine MAGL inhibitors through a structure-based hybridization strategy combining key pharmacophoric elements from previously reported MAGL and FAAH inhibitors [92]. In particular, the novel scaffold merged the amide phenolic portion of earlier benzoylpiperidine MAGL inhibitors, known to establish a crucial hydrogen-bond network within the catalytic site of the enzyme, with the 2-(3-(piperidin-4-ylmethyl)phenoxy)-5-(trifluoromethyl)pyridine moiety derived from the FAAH inhibitor PF-3845 [93]. Within this set of analogues, compound **40** (Figure 7) displayed the highest inhibitory activity. Its optimized architecture features a *para*-fluoro substituent on the phenolic ring and a trifluoromethyl group positioned at C4 of the pyridine moiety, modifications that markedly enhanced the electronic distribution and binding complementarity within the MAGL catalytic site. As a result, **40** exhibited an IC_50_ value of 2.0 nM, together with excellent selectivity over FAAH (IC_50_ > 10 µM). The inhibitor maintained full activity in the presence of DTT, indicating no involvement of covalent interactions with cysteine residues. Moreover, pre-incubation and dilution experiments confirmed a fully reversible and competitive inhibition profile. Molecular modeling studies predicted for compound **40** a binding mode characterized by a slightly shifted orientation toward the entrance of the MAGL catalytic channel due to the absence of the ketonic carbonyl group typical of previously developed benzoylpiperidine derivatives. In this case, the single amide carbonyl group was proposed to establish the key hydrogen-bond interactions with the oxyanion hole residues Ala51 and Met123, while the phenolic group maintained a stable hydrogen bond with His121, thereby contributing to ligand stabilization within the binding site. Beyond its enzymatic profile, compound **40** demonstrated a significant antiproliferative effect in pancreatic cancer primary cultures, including PDAC2 and PDAC3, where it reduced cell viability at low-micromolar concentrations (IC_50_ values of 12.61 and 7.25 µM, respectively). Moreover, compound **40** promoted apoptotic signaling through caspase-3 activation, reduced the expression of the anti-apoptotic factor Bcl-2 and the migration-related protein matrix metalloproteinase-9 (MMP9) and increased the levels of human equilibrative nucleoside transporter 1 (*h*ENT1), the major transporter involved in gemcitabine uptake, thereby contributing to its synergistic interaction with gemcitabine. From a pharmacokinetic standpoint, **40** displayed favorable ADME properties, including good passive permeability, high plasma stability and an acceptable metabolic profile in human liver microsomes.

Building on these findings, in 2024 the same research group further expanded this chemical scaffold, leading to the identification of a novel generation of benzylpiperidine- and benzylpiperazine-based MAGL inhibitors [94]. Among the synthesized analogues, benzylpiperazine **41** (Figure 7) was identified as one of the most potent inhibitors, exhibiting a strong reversible inhibitory activity against *h*MAGL with an IC_50_ value in the low-nanomolar range (5.2 nM). Compared with previously developed compounds from the same research group, such as compound **40** (Figure 7), compound **41** is characterized by the presence of a piperazine ring replacing the piperidine scaffold, as well as by a sulfur atom instead of an oxygen atom linking the 4-trifluoromethylpyridine moiety to the rest of the molecule. The inhibitory profile of **41** was consistently confirmed in cell-based assays, where the compound efficiently reduced MAGL activity in intact U937 cells, indicating adequate cellular permeability and effective target engagement. In parallel, selectivity studies showed that **41** displayed minimal activity against FAAH and related serine hydrolases, supporting a selective interaction within the endocannabinoid system. Molecular modeling studies suggested that the piperazine core enabled the formation of a stable water-bridged hydrogen bond between the positively charged nitrogen atom of compound **41** and the backbone carbonyl oxygen of Leu241. This interaction promoted a slight inward bending and consequent repositioning of the diarylsulfide fragment within the MAGL binding cavity, thereby altering its interaction pattern compared to its benzoylpiperidine analogue. From a drug-likeness perspective, compound **41** exhibited a favorable in vitro ADME profile, including good metabolic stability in human liver microsomes and human plasma, increased aqueous solubility and reduced membrane retention rate. Moreover, benzylpiperazine **41** exhibited a favorable safety profile, as indicated by a high LD_50_ value in a *Tenebrio molitor* coleoptera toxicity model. Altogether, these properties highlight **41** as a well-balanced reversible MAGL inhibitor combining potent enzymatic and cellular activity with selectivity and suitable pharmacokinetic features, thereby representing a valuable lead compound for further optimization.

In the same year, Di Stefano and colleagues reported the identification of a novel MAGL inhibitor by applying an original receptor-based computational approach named *Watermelon* [95]. This strategy, developed to generate pharmacophore models relying exclusively on the three-dimensional structure of the target protein, enabled the mapping of key interaction hotspots within the MAGL catalytic cavity using small molecular fragments as probes, whose most stable interactions were identified and translated into pharmacophoric features for virtual screening. Through the application of this workflow, compound **42** (Figure 7) was identified as the most active hit and experimentally confirmed as a reversible MAGL inhibitor, displaying an IC_50_ value of 14.6 µM. Enzymatic assays, performed in the presence of the reducing agent DTT and under different pre-incubation times, excluded covalent binding mechanisms, while kinetic studies supported a competitive mode of inhibition. These findings were further corroborated by molecular modeling studies, which showed that **42** establishes stable interactions within the MAGL binding site. In particular, **42** establishes persistent hydrogen-bond interactions with the oxyanion hole residues Ala51 and Met123 through its carbonyl group, with these contacts being maintained for most of the simulation time. In addition, the ligand displayed a face-to-face π-π stacking interaction with Tyr194, while its ethyloxycarbonyl moiety occupied a hydrophobic pocket lined by Ala151, Phe159, Leu148, Leu213, and Leu214. Notably, a water-mediated hydrogen-bond interaction involving the piperazine nitrogen of **42** and the backbone carbonyl of Ala51 was also observed. Beyond its enzymatic inhibitory profile, **42** was also investigated for its ability to modulate the antioxidant response. In cell-based assays, the compound induced a significant activation of the Nrf2 pathway at micromolar concentrations, without affecting cell viability. Overall, these results highlight **42** as a MAGL inhibitor combining reversible enzymatic inhibition with a promising antioxidant profile, supporting the potential of the *Watermelon* approach for the identification of biologically relevant MAGL modulators.

A more recent study from the same research group reported the design, synthesis, and preliminary biological evaluation of the first glycoconjugated benzoylpiperidine MAGL inhibitors [96]. The design rationale of these molecules was to functionalize the benzoylpiperidine core, in line with previously developed analogues by the same group [90,91], with a sugar moiety, specifically a glucopyranose unit. This strategy was intended to enhance cellular uptake in cancer cells by exploiting the overexpression of glucose transporters (GLUTs) observed in several tumor types. The glycoconjugated benzoylpiperidine **43** (Figure 7) inhibited *h*MAGL with an IC_50_ value of 68.8 µM. Although its potency in biochemical assays was lower than that of previously reported benzoylpiperidine MAGL inhibitors, very encouraging results were obtained in cell-based assays. In particular, compound **43** exhibited significant inhibitory activity against the PANC-1 pancreatic cancer cell line, with a GI_50_ value of 12 µM under low-glucose conditions, whereas no detectable activity was observed in a physiological pulmonary fibroblast cell line, thus supporting the selectivity of these newly developed MAGL inhibitors. While the mechanism of inhibition of the glycoconjugated derivatives was not investigated through dilution or pre-incubation assays, compound **43** retains the benzoylpiperidine pharmacophore, a scaffold whose reversible inhibition of MAGL has been extensively demonstrated in previous studies by the same research group through dedicated mechanistic investigations. Therefore, while experimental confirmation is still required, it is reasonable to hypothesize that compound **43** also acts as a reversible MAGL inhibitor. Further pharmacological investigations will be necessary to better elucidate the potential role of GLUT-mediated uptake in the cellular internalization of these compounds. Nonetheless, this study represents an important step toward the development of the first glycoconjugated inhibitors specifically designed to target MAGL.

In 2023, Jiang and colleagues reported the discovery of LEI-515 (compound **44**, Figure 7), a novel and reversible MAGL inhibitor designed to achieve strong peripheral activity while minimizing central side effects [97]. LEI-515 (**44**) was identified through a large high throughput screening (HTS) of approximately 234,000 molecules, followed by enzymatic assays and ABPP experiments. One compound emerged as the most promising lead and was then optimized through a medicinal chemistry program, during which more than 100 derivatives were synthesized. Throughout this process, the authors noticed that the ester functionality was crucial for potency, but it also represented a metabolic liability. For this reason, they replaced the ester with a *α*-CF_2_ ketone, which acts as an electrophilic moiety capable of forming a covalent yet reversible interaction with the catalytic serine of MAGL. This chemical modification ultimately led to the identification of the abovementioned LEI-515 (**44**). To assess reversibility, brain membrane proteosomes were pre-incubated with LEI-515 (**44**) and residual MAGL activity was monitored over time. The time-dependent recovery of the enzyme activity demonstrated that LEI-515 (**44**) inhibits MAGL through a reversible mechanism with a pIC_50_ value of 9.3, corresponding to an IC_50_ value of approximately 0.5 nM. Crystallographic analysis confirmed that LEI-515 (**44**) binds in the active site of MAGL as a deprotonated hemiketal, mimicking the enzymatic tetrahedral intermediate and stabilized by hydrogen bonds with residues of the oxyanion hole, proving a structural rationale for its high potency. The authors further validated LEI-515 (**44**) through an expanded pharmacological and in vivo characterization [98]. They confirmed the excellent selectivity of the compound for MAGL over other members of the serine hydrolase family and pharmacokinetic and tissue distribution studies demonstrated that it had minimal brain exposure and acted mainly in peripheral tissues, as reflected by the increase of 2-AG levels in the intestine, lung and liver, but not in the brain. The authors demonstrated strong efficacy in multiple in vivo models, including inflammatory and neuropathic pain as well as liver injury, without inducing tolerance, physical dependence or CB1-mediated central side effects. Overall, these studies propose LEI-515 (**44**) as a well-validated reversible MAGL inhibitor, peripherally restricted and with safe therapeutic activity in vivo.

In 2023, Xiang and collaborators evaluated the therapeutic effect of MAGL inhibitor M-18C (also named MAGLZ-18C, compound **45**, Figure 7) on acute kidney injury (AKI), a severe complication of various diseases for which effective treatments remain limited [99]. Considering the tissue distribution of MAGL, including kidneys, and the anti-inflammatory properties of MAGL inhibitors, the authors hypothesized that M-18C (**45**) may have enhanced potential for anti-inflammatory effects and protection against AKI. It should be noted that the original study did not disclose the design or synthetic route of M-18C (**45**), and no such information could be found in patents or other publications. They only mentioned that the compound (purity > 98%) was commercially obtained from Shandong New Time Pharmaceutical Co. LTD. (Linyi, China). M-18C (**45**) is characterized by a piperidine core substituted at the 4-position with an *N*-(3-chlorophenyl)carboxamide and at the 1-position with a methylindoline-2-carboxy group, and it inhibited *h*MAGL with an IC_50_ value of 662.6 nM. In vitro and in vivo investigations demonstrated that M-18C (**45**) exerted protective effects in LPS-induced AKI through the attenuation of renal tissue damage, reinforcement of the intestinal barrier and regulation of gut microbial communities and serum metabolites, highlighting its potential as promising candidate for further exploration. Three years later, the same research group investigated the anti-inflammatory activity and lipid-metabolism regulatory effects of M-18C (**45**) in a LPS-induced sepsis-associated liver injury (SALI) mice model [100]. SALI is a severe form of liver damage associated with sepsis, a systemic hyper inflammatory condition characterized by high mortality rates. Given the urgent need for effective therapeutic strategies for SALI, together with the well-known anti-inflammatory properties of MAGL inhibitors, the authors explored whether M-18C (**45**) could exert protective effects in this pathological condition. The study revealed that treatment with M-18C (**45**) significantly alleviated liver injury in LPS-treated mice. The hepatoprotective effects were associated with a marked reduction in pro-inflammatory cytokines together with an increase in interleukin-10 (IL-10). In addition, M-18C (**45**) contributed to the preservation of mitochondrial structure and function, modulated the transforming growth factor *β*/small mother against decapentaplegic (TGF-*β*/Smad) signaling pathway and restored fatty acid metabolism. Altogether, these findings demonstrate that MAGL inhibitor M-18C (**45**) may represent a potential pharmacological candidate for the treatment of sepsis-related complications. Importantly, this piperidine-based compound can be classified as a reversible MAGL inhibitor, as reported by the authors in their most recent work published in 2026 [100]; however, the inhibition mechanism was not supported by experimental pre-incubation, dilution or other mechanistic assays.

In 2024, Yu and co-workers described a series of naphthyl amide derivatives as a novel class of reversible MAGL inhibitors, identifying the *cis*-2,3-dimethylpiperazine derivative ±**46** (Figure 7) as the most potent and selective compound of this series [101]. Structurally, compound ±**46** is characterized by an *α*-naphthyl amide core linked to a *cis*-2,3-dimethyl-substituted piperazine, a configuration that preserves the amide carbonyl group required for H-bond interactions within the MAGL oxyanion hole while optimizing hydrophobic and π-π interactions within the enzyme binding pocket. Consistent with these structural features, ±**46** emerged as the most active compound in a colorimetric assay against recombinant *h*MAGL, exhibiting a pIC_50_ value of 7.1. The reversible mode of inhibition was demonstrated by dilution assays, which showed a proportional loss of inhibitory activity upon compound dilution, as well as by pre-incubation experiments, in which no time-dependent increase in inhibition was observed. The potency and selectivity of ±**46** toward endogenous MAGL were further assessed by ABPP experiments using mouse brain membrane proteomes (*m*MAGL), where the compound produced more than 60% reduction in *m*MAGL activity at 100 µM while maintaining a favorable selectivity profile over other serine hydrolases, such as ABHD6. In addition to its enzymatic activity, ±**46** displayed significant antiproliferative effects in multiple cancer cell lines, including H460 (lung cancer), HT-29 and CT-26 (colon cancer) and Huh7 and HCCLM-3 (hepatocellular cancer), with pIC_50_ values greater or equal to 5.0; among these, H460 cells were the most sensitive, with a pIC_50_ of 6.2. Importantly, ±**46** exhibited minimal cytotoxicity in normal human cell lines (i.e., BEAS-2B immortalized human bronchial epithelial cell line).

One year later, the same research group identified a novel and potent reversible MAGL inhibitor based on the previously developed naphthyl amide piperazine scaffold [102]. Starting from compound ±**46** [101], they carried out a systematic medicinal chemistry optimization focused on two main regions of the molecule: (1) the naphthalene moiety, which occupies the hydrophobic pocket of the enzyme, and (2) the phenyl group linked to the piperazine nitrogen. SAR studies clearly demonstrated that the naphthalene ring is essential for activity, as its replacement or reduction resulted in a strong loss of potency, thus it was maintained fixed. Conversely, the other side of the molecule was optimized by introducing a linker between the phenyl and the piperazine ring, leading to the identification of compound **47** (Figure 7), a naphthyl amide piperazine with a *meta*-chloro substituted phenyl group, as the most potent inhibitor from the series. Compound **47** inhibited *h*MAGL with high potency, exhibiting a pIC_50_ of 8.0 in enzymatic assays. The authors performed a dilution and pre-incubation assay to assess reversibility of compound **47**: the inhibition decreased proportionally after a 10-fold dilution, whereas no time-dependent increase in inhibition was observed when the enzyme was pre-incubated before the addition of the substrate, confirming that compound **47** acts as reversible MAGL inhibitor. To assess the selectivity profile of the newly developed MAGL inhibitor **47**, the authors tested it in mouse brain membrane preparations by using ABPP. Compound **47** effectively inhibited MAGL, showing only limited activity toward other serine hydrolases. Furthermore, compound **47** reduced proliferation, induced apoptosis and inhibited migration in colon cancer cells HT-29. Taken together, these results identify the naphthyl amide piperazine scaffold as a promising chemical feature for the development of new selective and reversible MAGL inhibitors.

##### Spiro Carbamates

In 2021, Ikeda and colleagues developed a series of spiro carbamates as reversible MAGL inhibitors, identifying compound **48** (Figure 6) as the most potent derivative [103]. Starting from an initial HTS hit, the authors applied a structure-based drug design approach, supported by co-crystals structures to understand the key interactions within the MAGL active site. They identified the central amide carbonyl group as a key anchoring element within the oxyanion hole, where it establishes hydrogen bonds with Ala51 and Met123. Based on these findings, the authors designed compound **48**, characterized by a central amide carbonyl group bearing, on one side, a 7-oxa-5-azaspiro [*3.4*]octan-6-one spirocyclic ring, which preserves the key interactions with His121 and Arg57 while improving lipophilic ligand efficiency (LLE), and, on the other side, a 3-chloro-4-methylbenzyloxy moiety directly linked to the azetidine ring of the amide scaffold. Spiro carbamate **48** combined a low nanomolar MAGL inhibition (IC_50_ = 6.2 nM), high selectivity over FAAH and ABHD6, a low hERG liability (18.1% at 10 μM), good oral exposure and effective brain penetration in mice after oral dosing. Surface plasmon resonance (SPR) experiments revealed a slow dissociation rate (*K*_off_ = 0.0036 s^−1^) and a short binding half-life (t_1/2_ = 3.3 min), indicating a non-covalent binding for compound **48**. The LLE also improved compared to the initial hit, from 4.5 to 5.9 in compound **48**. The reversible mechanism of inhibition was also confirmed by a dilution assay, in which MAGL activity was fully restored after reducing the inhibitor concentration. Moreover, compound **48** produced the expected in vivo pharmacodynamic effects, increasing brain 2-AG levels and decreasing arachidonic acid levels. Three years later, Arimura and colleagues reported the in vivo pharmacological characterization of compound **48**, to evaluate whether this reversible MAGL inhibitor could still provide meaningful neuroprotective effects [104]. After oral administration in mice, compound **48** rapidly reached the brain, with peak concentrations after 1 h, producing a robust, dose-dependent increase in brain 2-AG levels together with a reduction in AA levels. Target engagement studies demonstrated that 99% of MAGL occupancy was achieved at 1 mg/kg, demonstrating a good oral bioavailability leading to an efficient inhibition in the CNS. Using kainic acid (KA)-induced neurodegeneration model, the authors showed that **48** significantly suppressed neuroinflammatory responses, reducing neuronal cell loss in the hippocampus and improving cognitive performance. To investigate how compound **48** elicits its neuroprotective effects, the authors tested whether these effects depend on cannabinoid receptor activation: co-administration of **48** with either a CB1 or CB2 receptor antagonist reduced the protective effects although these were not completely abolished. Gene expression and pathway analyses in the hippocampus showed that the compound normalized inflammation and neurotransmission altered by neurodegeneration. Overall, the authors presented compound **48** as a reliable and well-characterized reversible MAGL inhibitor, with a balanced pharmacological profile that combines strong brain target engagement and neuroprotective effects. These properties support its use as a valuable tool compound to further investigate reversible MAGL inhibition in vivo and explored its potential application in CNS-related disorders, including neurodegenerative diseases.

##### cis-Hexahydro-Pyrido-Oxazinones

In 2024, Kuhn and colleagues reported a novel series of reversible MAGL inhibitors starting from a focused screening of a subset of approximately 71,100 compounds from the Roche compound library, selected through an integrated approach combining diverse lead-like subsets, computationally selected compounds, and compounds derived from a previous project targeting different lipases [105]. Enzymatic screening yielded 2398 hits with IC_50_ values below 10 μM (hit rate: 3.4%). Among these, several benzoxazinone-based compounds exhibited high inhibitory potency and favorable ligand efficiency. A decisive improvement was achieved replacing the phenyl ring of the benzoxazinone head group with saturated piperidine, yielding a hexahydropyrido-oxazinone (HHPO) scaffold. In this series, the *cis*-(4*R*,8*S*) HHPO derivative **49** (Figure 6) was identified as the most promising compound, showing high potency on *h*MAGL (IC_50_ = 32 nM), while displaying markedly improved physicochemical properties. Compound **49** showed a significantly increased aqueous solubility (231 μg/mL), a low *log*D_brain_ of 1.8 in lipid membrane binding assay (LIMBA), a human plasma free fraction of 4.3%, high permeability through membranes (PAMPA P_eff_ = 12.8 cm/s*10 × 10^−6^) and stability across a pH range of 1–10 in aqueous buffer systems. SPR studies confirmed a reversible, non-covalent binding mode, with a *K*_d_ value of 45 nM for *h*MAGL and of 64 nM for *r*MAGL, accompanied by rapid association and dissociation kinetics. Compound **49** demonstrated excellent selectivity, showing no detectable activity on a panel of 50 off-targets or related serine hydrolases (ABHD6, ABHD12, FAAH, and DGL) at concentrations up to 10 μM in ABPP assays. Favorable absorption, distribution, metabolism, and excretion (ADME) properties were observed, including high microsomal and hepatocellular stability, low in vivo clearance in rats (4.6 mL/min/kg, corresponding to approximately 10% of liver blood flow), a moderate volume of distribution (*V*_ss_ = 1.3 L/kg) and a terminal half-life of 5.1 h. After intraperitoneal and oral administration, **49** achieved excellent systemic and brain exposure, with total brain-to-plasma ratio of approximately 1. The ratio of unbound brain concentrations (estimated from cerebrospinal fluid levels) to unbound plasma concentrations (corrected for plasma protein binding) reached values up to 0.53, without adverse behavioral effects in rodent pharmacokinetic studies. In in vivo target engagement studies, **49** induced a dose-dependent increase in brain of 2-AG levels and a concomitant reduction in AA, reaching a target occupancy of 79% at 10 mg/kg and almost complete occupancy at 30 mg/kg. Furthermore, in LPS murine model of neuroinflammation, **49** significantly prevented LPS-induced mRNA expression of the pro-inflammatory markers interleukin-1*β* (IL-1*β*) and lipocalin-2 (LCN2) specifically in brain. Collectively, these findings identify compound **49** as a highly promising CNS-directed MAGL reversible inhibitor, combining favorable brain exposure and target engagement with effective modulation of neuroinflammatory pathways.

##### Anilides

Very recently, Bononi and co-workers reported a novel series of *ortho*-hydroxyanilide derivatives as reversible MAGL inhibitors [106]. Within this library, the phenylbutanamide analogue **50** (Figure 6) emerged as the most potent compound, displaying submicromolar inhibitory activity (IC_50_ = 0.34 µM) and a competitive, fully reversible mechanism of action. Molecular modeling studies indicated that the 2-fluorophenol fragment of compound **50** engages the MAGL oxyanion hole through hydrogen bonds with the backbone NH groups of Ala51 and Met123 and with the hydroxyl side chain of Ser122, thereby contributing to the proper stabilization and anchoring of the ligand within the binding site. The biphenyl and phenylpropyl moieties of the ligand further stabilize the complex by establishing extensive hydrophobic contacts with residues such as Ala151, Ala156, Phe159, Leu208, Ala216 and Val217, supporting the predicted binding mode. Compound **50** also exhibited an improved selectivity profile, showing only moderate inhibition of FAAH and negligible affinity for CB1 and CB2 receptors. Beyond its enzymatic activity, this derivative demonstrated the ability to activate the Nrf2 antioxidant pathway at submicromolar concentrations and to attenuate IL-1*α*-induced NFκB activation, suggesting potential relevance in pathological conditions where oxidative stress and inflammation are prominent, including neurodegenerative diseases. From an ADME perspective, compound **50** displayed good passive permeability across gastrointestinal and BBB models, together with excellent plasma stability. However, its metabolic stability in human liver microsomes was limited, with extensive degradation attributed to cleavage of the amide bond. Overall, compound **50** represents a promising lead for the development of MAGL inhibitors with ancillary antioxidant and anti-inflammatory properties, although further optimization is required to improve its microsomal stability.

### 2.3. MAGL Inhibitors with Undefined Mechanism of Inhibition

This section focuses on natural and synthetic compounds that have been identified as MAGL inhibitors, for which the precise mechanism of inhibition, whether reversible or irreversible, has not yet been fully elucidated. In particular, compounds were included in this section when no experimental evidence from mechanistic assays (such as dilution or pre-incubation studies, DTT sensitivity tests, mass spectrometry-based investigations or related approaches) was available to support a definitive classification as reversible or irreversible inhibitors, or when the available data were inconclusive. In contrast to the classes of irreversible and reversible inhibitors discussed above, where natural products are relatively underrepresented compared to synthetic small molecules, compounds with an undefined mechanism of MAGL inhibition appear to be more evenly distributed between natural and synthetic compounds. This reflects the fact that many studies reporting MAGL inhibition, particularly in the context of natural product research, primarily focus on the identification of bioactive compounds, while detailed mechanistic characterization is often not yet available. The main examples reported in the literature, from 2020 to 2026, are summarized in the following sections.

#### 2.3.1. Natural Compounds

Natural products have emerged as a valuable source of structurally diverse bioactive molecules with the potential to modulate key enzymatic targets involved in disease progression. Although detailed mechanistic characterization is often lacking, these works discussed herein highlight the promise of natural compounds as starting points for the development of new MAGL-targeting therapeutic strategies in a variety of pathological conditions, including inflammation, pain, cancer and metabolic disorders.

##### Alkaloids

**Figure 8:**
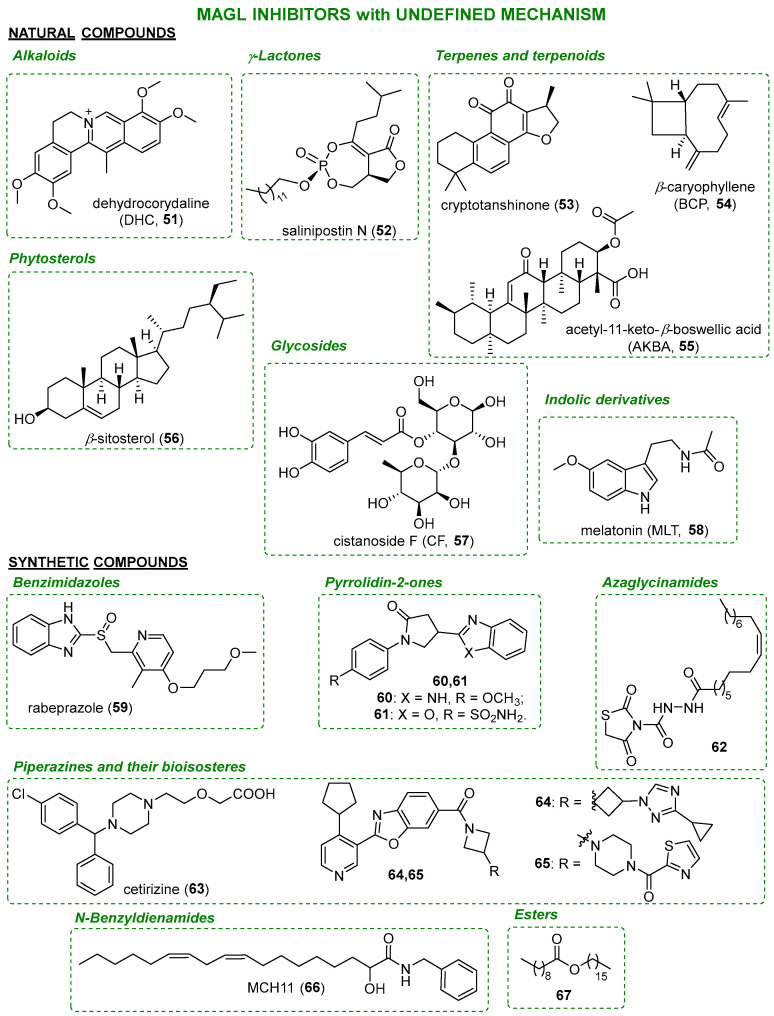
Natural and synthetic MAGL inhibitors with undefined mechanism of inhibition.

In 2022, Mei and colleagues screened bioactive compounds from herb extracts of traditional Chinese medicine (TCM) using a yeast-based drug discovery strategy [107]. This ligand-fishing approach led to the identification of dehydrocorydaline (DHC, compound **51**, Figure 8), a quaternary ammonium alkaloid isolated from *Corydalis rhizoma* extract, as a promising MAGL inhibitor. Indeed, when evaluated in vitro and co-incubated with MAGL-displaying yeast cells and non-displaying control yeast cells, DHC (**51**) exhibited an IC_50_ value of 240.1 µM. In addition, molecular docking analyses suggested the formation of hydrogen bonds between the two methoxy groups of DHC (**51**) and Asn153 of MAGL, which may contribute to the binding of DHC (**51**) within the enzyme active site. Given the long use of *Corydalis rhizoma* in TCM for pain relief, this alkaloid was also evaluated in vivo in chronic constriction injury (CCI) rats, a widely used animal model of chronic neuropathic pain. In this model, DHC (**51**) significantly alleviated mechanical and thermal pain hypersensitivity by suppressing MAGL activity, thereby inhibiting 2-AG degradation.

##### γ-Lactones

In 2022, Kudo and co-workers developed an efficient screening strategy aimed at the identification of novel phosphotriester compounds exhibiting inhibitory activity against serine hydrolases [108]. This liquid chromatography-tandem mass spectrometry (LC-MS/MS)-based approach was applied to *Salinispora tropica* CNB-440, a marine actinomycete known to produce salinipostins (SPTs), enabling the identification of three previously unreported SPT analogues. Among these new derivatives, salinipostin N (**52**, Figure 8), characterized by a long-chain bicyclic enol-phosphotriester ring bearing an *iso*-pentyl substituent at C6 position, displayed a nanomolar inhibitory activity toward *h*MAGL in a colorimetric in vitro assay, with an IC_50_ value of 178 nM. The development of this efficient LC-MS/MS-based screening method was confirmed as a valuable strategy to foster the discovery of previously unreported natural phosphotriester compounds, which could act as potent MAGL inhibitors.

##### Terpenes and Terpenoids

As mentioned in previous sections, in parallel with the identification of selective MAGL inhibitors, considerable attention has been directed toward the development of reliable biochemical assays capable of accurately measuring MAGL enzymatic activity [54]. In this context, in 2022 Deng and co-workers reported the synthesis of a novel fluorogenic substrate, 6-hydroxy-2-naphthaldehyde-arachidonate (AA-HNA) and established an AA-HNA-based assay that enables the rapid identification of MAGL inhibitors [109]. Importantly, the integration of this assay with ABPP allowed the efficient screening of a library of 320 naturally derived organic compounds with potential therapeutic relevance. This screening campaign led to the identification of several classes of MAGL inhibitors, including flavonoids, diterpenes, and *β*-carbolines. Among the active compounds, the quinoid diterpene cryptotanshinone (compound **53**, Figure 8) emerged as the most promising candidate and was therefore selected for further investigation. Cryptotanshinone (**53**) is a natural product isolated from the roots of *Salvia miltiorrhiza*, a medicinal plant widely used in traditional medicine and has previously been reported to exert multiple beneficial biological effects, including neuroprotective, anti-fibrotic, anti-inflammatory and anti-cancer activities [110,111]. In the AA-HNA-based enzymatic assays, cryptotanshinone (**53**) effectively inhibited MAGL, displaying a pIC_50_ value of 4.9. Its selectivity profile was further investigated toward ABHD6 and ABHD12, and both substrate-based and ABPP assays suggested that compound **53** preferentially targets MAGL while displaying lower activity against ABHD6 and ABHD12. Competitive ABPP experiments performed on endogenous mouse membrane proteomes revealed that compound **53** exhibited significant inhibitory potency toward recombinant *h*MAGL and retained moderate activity against endogenous mouse MAGL (approximately 50–60% inhibition); moreover, cryptotanshinone (**53**) behaves as a relatively broad-spectrum serine hydrolase inhibitor. Furthermore, the antiproliferative properties of compound **53** were subsequently evaluated in a panel of cancer cell lines, including colon (HT-29), ovarian (OVCAR-3), lung (A549, H1975, HCC827), melanoma (B16-F10), cervical carcinoma (HeLa) and epidermal carcinoma (A431). Cryptotanshinone (**53**) exhibited pronounced cytotoxic activity across all tested cell lines, with pIC_50_ values greater than or equal to 5, suggesting an extensive antitumor potential. However, comparison with the reference MAGL inhibitor KML29 (compound **11**, Figure 3A) [42], which showed antiproliferative activity only in B16-F10 and HT-29 cells, suggested that the cytotoxic profile of compound **53** may involve additional off-target effects beyond MAGL inhibition, likely contributing to its broader anticancer activity. Finally, molecular docking studies were performed to elucidate the putative binding mode of ligand **53** within the MAGL active site. The simulations suggested that the carbonyl group at the C11 position of cryptotanshinone (**53**) may engage in a hydrogen-bond interaction with the side chain of Arg57. In addition, the aromatic ring was predicted to establish π-π stacking interactions with Tyr194. The dimethylcyclohexane moiety was located within a hydrophobic pocket formed by residues such as Leu241 and Ala51, whereas the methyl-2,3-dihydrofuran moiety occupied the hydrophobic region delimited by Val270, Lys273, Val191, and Glu190. Overall, this work highlights the effectiveness of combining fluorogenic substrate assays with ABPP as a powerful platform for the expedited discovery of biologically relevant MAGL inhibitors.

Lately, Klawitter and collaborators investigated the mechanism underlying the analgesic effects of *β*-caryophyllene (BCP, *trans*-*β*-caryophyllene, compound **54**, Figure 8), a highly volatile and lipophilic sesquiterpene abundant in the essential oils of various culinary and medicinal plants, including rosemary, *Cannabis sativa*, clove, and hops [112]. Despite its approval by the Food and Drug Administration (FDA) and the European Food Safety Authority (EFSA) for use as an additive in food and cosmetic products, as well as its well-established safety profile and reported therapeutic potential in several conditions such as chronic pain, inflammation and cancer, the precise mechanism underlying the analgesic effects of BCP (**54**) remains poorly understood. The scientific literature has reported conflicting evidence regarding its interaction with cannabinoid receptor type 2 (CB2), with early studies suggesting CB2 agonism [113], whereas more recent investigations have indicated weak or negligible binding affinity for this receptor [114,115,116]. In this study, the researchers found that BCP-induced analgesia may be partially attributed to elevated 2-AG levels resulting from MAGL inhibition, leading to enhanced endocannabinoid signaling and subsequent activation of cannabinoid receptors. Indeed, BCP (**54**) inhibited MAGL activity in vitro with an IC_50_ value of 15.8 µM and produced a dose-dependent increase in 2-AG concentrations in both plasma and spinal cord tissues in vivo.

Taken together, these findings identify MAGL inhibition as a previously unrecognized contributor to the analgesic properties of BCP (**54**), further supporting the potential role of MAGL inhibitors in pain management.

Another natural compound within the terpenoid class is acetyl-11-keto-*β*-boswellic acid (AKBA, compound **55**, Figure 8), a major active compound isolated from the gum resin of *Boswellia serrata*, a plant long employed in TCM for its anti-inflammatory, antimicrobial, antitumor, antioxidant and anti-aging effects [117,118]. AKBA (**55**) has also demonstrated neuroprotective properties and is generally well tolerated in clinical applications, particularly in the management of osteoarthritis, a degenerative condition characterized by articular cartilage degradation for which safer and more effective therapies are urgently required [119,120]. Given its favorable safety and pharmacological profile, AKBA (**55**) has been proposed as a promising therapeutic candidate for nonalcoholic steatohepatitis (NASH), which represents the progressive and inflammatory form of nonalcoholic fatty liver disease (NAFLD), for which effective treatments remain limited. In this context, very recently, Luan and colleagues investigated the therapeutic potential of AKBA (**55**) in mouse models of NAFLD and investigated its underlying mechanism of action [121]. Their results demonstrated that AKBA (**55**) exerts protective effects in NAFLD, at least in part, through inhibition of MAGL activity, thereby modulating lipid metabolism and hepatic metabolic dysfunction. In vitro assays, using lysates from primary mouse hepatocytes overexpressing MAGL, highlighted a direct interaction of AKBA (**55**) with the enzyme, exhibiting a high binding affinity (dissociation constant *K*_d_ = 4.06 × 10^−5^ mol/L). In addition, molecular docking analyses disclosed the putative binding mode of AKBA (**55**) within the MAGL active site, highlighting key interactions with residues Glu60, Met64, Thr279, and Phe283; however, the specific structural features of AKBA responsible for these interactions were not detailed in the study. Importantly, mutations in these residues were shown to impair enzymatic activity, suggesting that they are critical for MAGL’s catalytic function. In summary, these results emphasize AKBA’s potential to improve NAFLD by inhibiting MAGL, highlighting both its mechanism of action and the therapeutic value of targeting MAGL in metabolic liver disorders.

##### Phytosterols

In 2024, Sadino and co-workers combined in silico and in vitro approaches to evaluate the phytoconstituents of *Morinda citrifolia*, a traditional Polynesian medicinal plant previously reported for its neuroprotective properties [122], for their potential ability to bind and inhibit *h*MAGL [123]. Nine bioactive compounds were identified in *M. citrifolia* fruit, including sterols (stigmasterol and *β*-sitosterol), lignans (americanin), iridoid glycoside (asperuloside), anthraquinones (damnacanthal), flavonoids (quercetin and rutin), coumarin (scopoletin) and triterpenoids (ursolic acid), and were subsequently subjected to molecular docking studies toward MAGL. Among these compounds, *β*-sitosterol (compound **56**, Figure 8) emerged as the most promising candidate, as it was predicted to enter and occupy the catalytic site of MAGL while interacting with key residues. In detail, this phytosterol was found to reside within the hydrophobic pocket of the enzyme, establishing hydrophobic or van der Waals interactions with several residues, including Ser122, Cys242 and His269, which are known to be involved in MAGL catalytic activity. Moreover, molecular dynamic simulations disclosed that *β*-sitosterol (**56**) exhibited one of the most favorable predicted binding energies (−11.20 kcal/mol) and formed a highly stable *β*-sitosterol/MAGL complex. Finally, in vitro enzymatic assays on *h*MAGL confirmed the inhibitory activity of *β*-sitosterol (**56**) which displayed an IC_50_ value of 8.10 µg/mL. In conclusion, the phytosterol fraction of *M. citrifolia* fruit may contribute to its neuroprotective and anti-inflammatory effects through modulation of MAGL activity.

##### Glycosides

In 2025, Tan and collaborators, after demonstrating the anti-tumorigenic and anti-metastatic role of 2-AG signaling in bladder cancer (BCa) cells, screened a medicinal-food compound library comprising 514 compounds with the aim of identifying novel MAGL inhibitors with potential anti-cancer activity [124]. Among the screened compounds, Cistanoside F (CF, **57**, Figure 8), a phenylethanol glycoside isolated from *Cistanche herba*, was selected as promising candidate based on its previously reported biological properties, including anti-oxidant, anti-inflammatory and anti-tumor activities [125,126,127]. In vitro cellular assays demonstrated that CF (**57**) efficiently inhibited MAGL activity in SW780 and RT112 human bladder transitional cell carcinoma lines when tested at 4.0 and 8.0 nM, without altering cell viability, MAGL protein expression levels or the activity of other serine hydrolases. The precise mechanism underlying MAGL inhibition by CF (**57**) still remains undefined, as mechanistic investigations only proposed several possible modes of action, including competitive inhibition, irreversible mechanism and/or allosteric modulation. Importantly, in vivo experiments highlighted a synergistic effect of CF (**57**) in combination with 2-AG, leading to a notable reduction in both tumor growth and metastasis in BCa mice models.

##### Indolic Derivatives

Although the natural MAGL inhibitors discussed so far are mainly derived from plant sources, this category can be reasonably extended to include endogenous small molecules of natural origin. In this context, melatonin (MLT, compound **58**, Figure 8), an indole-based hormone biosynthesized in the pineal gland, represents an intriguing example of a non-plant natural compound with emerging modulatory activity toward MAGL. Melatonin’s main function is the regulation of circadian rhythms, but it has been widely reported to exert antioxidant, anti-inflammatory, immunomodulatory and antitumor effects, making it a molecule of increasing interest in cancer research [128,129,130]. In 2025, Li and colleagues investigated the involvement of MAGL in pancreatic cancer progression and explored MLT (**58**) as a potential endogenous MAGL inhibitor [131]. In this study, MLT (**58**) was shown to significantly downregulate MAGL gene expression in PANC-1 human pancreatic cancer cell line, confirming its ability to modulate MAGL activity at the transcriptional level.

#### 2.3.2. Synthetic Compounds

##### Benzimidazoles

In 2020, Dahabiyeh and co-workers investigated the unknown mechanism of action of three proton pump inhibitors (PPIs), rabeprazole, lansoprazole and pantoprazole, underlying their ability to regulate glucose-insulin homeostasis [132]. The authors hypothesized that the beneficial effect of PPIs on glycemic control in type 2 diabetes could be ascribed to MAGL inhibition, considering the crucial role of this enzyme in lipolysis and inflammatory processes. The inhibitory activity of the three drugs on *h*MAGL was evaluated through a colorimetric in vitro assay, with rabeprazole (compound **59**, Figure 8) exerting the best inhibitory activity, displaying an IC_50_ value of 4.2 µM. In addition, rabeprazole (**59**) was successfully docked into MAGL active site (PDB code: 3PE6), using the site-feature docking algorithm LibDock, yielding a score of 148.44, which was comparable to that of reversible inhibitor ZYH (183.72, compound **18**, Figure 3A). Rabeprazole (**59**) formed five key hydrogen bonds with residues His272, Arg57, His121, Ser122, and Gly177. Specifically, the sulfoxide oxygen atoms interacted with the imidazole side chain of His272 through a water-mediated contact, while the benzimidazole *sp*^2^ nitrogen atoms formed an additional water-bridged interaction with the guanidinium group of Arg57. Furthermore, the *sp*^2^- hybridized nitrogen of the methylpyridine linker interacted with His121, the aromatic oxygen atoms engaged the hydroxyl side chain of Ser122 and the terminal methoxy group formed a hydrogen bond with the backbone carbonyl of Gly177. In addition, the central methylpyridine moiety established hydrophobic interactions with Leu184. Overall, these results support the hypothesis that PPIs may regulate glycemic control in type 2 diabetes through MAGL inhibition.

##### Pyrrolidin-2-Ones

In 2021, Altamimi and colleagues designed and synthesized benzothiazole- and benzimidazole-linked pyrrolidin-2-ones as potential MAGL inhibitors [133]. Among the eighteen developed compounds, the benzimidazole-linked pyrrolidin-2-one derivatives showed the highest inhibition activity against *h*MAGL. In particular, benzimidazole **60** (Figure 8), characterized by a 4-methoxyphenyl substitution on the nitrogen atom of the pyrrolidin-2-one ring, was the most potent and selective MAGL inhibitor of this series, with an IC_50_ value of 9.4 nM on MAGL versus 52 µM for FAAH. Compound **60** was further investigated to better elucidate its binding pose within the MAGL active site. Molecular docking studies revealed that the carbonyl group is located in the oxyanion hole, forming three key hydrogen bonds with Ala51, Met123 and Ser122; the benzimidazole core resides in the long lipophilic channel, establishing π-π stacking interactions with Tyr192; and finally, the 4-methoxyphenyl ring engages in van der Waals interactions with Leu148, Leu213 and Leu241. The physiochemical and pharmacokinetic properties of **60**, predicted by using the prediction QikProp program, fell within the optimal range for orally bioactive CNS drugs. The therapeutic relevance of benzimidazole-linked pyrrolidin-2-one **60** was additionally supported by in vivo antinociceptive studies. In the formalin-induced nociception test, a well-established animal model of neuropathic pain, compound **60**, produced a dose-dependent attenuation of pain response in both the acute and late phases, indicating a dual peripheral and central modulatory action. Notably, oral administration at 30 mg/kg significantly reduced the pain response and demonstrated greater efficacy than the reference drug gabapentin (GBP).

Building on these results, the following year the same research group reported a new series of benzoxazole-linked pyrrolidin-2-ones as potential MAGL inhibitors [134]. Among these derivatives, benzoxazole **61** (Figure 8) bearing a 4-sulfamoylphenyl substituent on the nitrogen atom of the pyrrolidin-2-one ring, emerged as the most potent analogue with an IC_50_ value of 7.6 nM, surpassing even the parent compound **60** (IC_50_ = 9.4 nM) [133]. The selectivity of **61** for MAGL over FAAH was confirmed by in vitro enzymatic assay (IC_50_ = 68 µM). Moreover, pyrrolidin-2-one **61** displayed physicochemical and predicted pharmacokinetic properties comparable to those of compound **60**, along with a similar binding mode within the MAGL active site, as indicated by computational studies. Evaluation with the admetSAR 2.0 web tool [135] further supported its favorable drug-like profile. Specifically, compound **61** was predicted to possess high BBB permeability and strong human intestinal absorption. It was also classified as non-mutagenic, non-carcinogenic and less toxic than its 4-nitrophenyl substituted analogue, showing and estimated LD_50_ value of 2.21 mol/kg. Moreover, in the formalin-induced nociception test, pyrrolidin-2-one **61** remarkably attenuated the pain response both in acute and late phases in a dose-dependent manner; in addition, it displayed greater potency than the positive control GBP at 30 mg/kg. Finally, in vitro antiproliferative screening assays on a panel of 60 cell lines of nine types of cancers, revealed that MAGL inhibitor **61** exerted a notable anticancer activity, particularly against the SNB-75 CNS cancer cell line (percentage of growth inhibition %GI = 31.88), the UO-31 renal cancer cell line (%GI = 29.95) and the MDA-MB-231/ATCC breast cancer cell line (%GI = 31.88).

##### Azaglycinamides

Considering that aza-analogues of glycine-containing peptides are widely exploited in the treatment of neurodegenerative diseases and cancer, in 2021 Shaik and co-workers designed and synthesized a new class of heterocyclic azaglycinamides as potential MAGL inhibitors, functionalized at both the *N*-terminal and *C*-terminal azaglycinyl moieties [136]. The compounds incorporated different heterocyclic motifs at the *C*-terminal position, including pyrrolyl, imidazolyl, and thiazolidine-2,4-dione rings. Among these series, the thiazolidine-2,4-dione derivatives displayed the most promising inhibitory activity against MAGL. In particular, azaglycinamide **62** (Figure 8) emerged as the most active compound, exhibiting an IC_50_ value of 21.05 µM on the isolated enzyme. Structurally, compound **62** bears a thiazolidine-2,4-dione heterocycle at the *C*-terminal azaglycinyl moiety and a *N*-oleoyl chain at the *N*-terminal position, two structural features that are proposed to enhance hydrogen-bonding and hydrophobic interactions within the MAGL binding pocket, as supported by molecular docking studies. In addition, compound **62** also exerted the highest cytotoxic activity against the MCF-7 human breast cancer cell line (IC_50_ value of 41.13 µM) compared to its corresponding *C*-pyrrolyl and *C*-imidazolyl analogues. Furthermore, antinociceptive studies highlighted the effectiveness of azaglycinamides **62** in attenuating inflammation-induced pain behaviors. It should be noted that no assays were performed to determine the mechanism of MAGL inhibition by azaglycinamide **62**, which is why it is classified here among inhibitors with an undefined mechanism of inhibition (Section 2.3). However, the authors highlighted the structural similarity of **62** to maleimide-based scaffolds, which feature a five-membered ring containing a nitrogen atom and a dione function, and it can be reasonably hypothesized, by analogy, that **62** may act as an irreversible MAGL inhibitor.

##### Piperazines and Their Bioisosteres

In 2023, Andrei and colleagues aimed to repurpose the therapeutic potential of the approved drugs cetirizine, an antihistaminic H1 receptor antagonist, and levetiracetam, an antiepileptic drug targeting the synaptic vesicle glycoprotein 2A (SV2A) [137]. Based on preliminary SAR analyses of known MAGL inhibitors, they selected cetirizine and levetiracetam to evaluate their potential as MAGL inhibitors in the treatment of osteoarthritis. Both compounds inhibited *h*MAGL in the micromolar range (cetirizine: IC_50_ = 9.39 µM, levetiracetam: IC_50_ = 3.01 µM); however, cetirizine (compound **63**, Figure 8) proved to be more promising than levetiracetam in in vivo experiments assessing anti-inflammatory and antinociceptive effects in animal models of osteoarthritis. Molecular modeling studies revealed that the two aromatic rings of cetirizine occupy the long hydrophobic channel of MAGL, establishing π-π interactions with the hydrophobic residues of the enzyme. The piperazinyl core of the molecule acts as a linker connecting the hydrophobic moiety to the carboxylic group, which is positioned in the oxyanion hole containing the catalytic triad and forming hydrogen bonds with Ser122 and Ala51. Based on molecular docking studies, it can be hypothesized that cetirizine (**63**) may act as a reversible MAGL inhibitor, as the carboxylic group forms a hydrogen bond with Ser122. However, in the absence of dedicated mechanistic studies, including assays to determine whether inhibition is competitive or non-competitive, the compound must be classified among MAGL inhibitors with an undefined mechanism of action (Section 2.3). In vivo studies demonstrated that cetirizine (**63**) alleviated both mechanical and thermal nociception in a rat model of osteoarthritis induced by complete Freund adjuvant (CFA). Cetirizine (**63**) also exhibited significant anti-inflammatory effects, reducing CFA-induced edema, inflammatory cell infiltration and granuloma formation in the affected paw. These results highlight the potential of cetirizine as a multitarget therapeutic agent, acting both as a MAGL inhibitor and an H1 receptor antagonist, capable of addressing the key pathological features of osteoarthritis pain and inflammation.

Very recently, Nippa and collaborators reported a machine-learning-based workflow aimed at accelerating the hit-to-lead optimization phase in the discovery of novel MAGL inhibitors [138]. Using a “machine-learning funnel”, a virtual library of 26,375 hypothetical compounds was progressively filtered to identify molecules displaying favorable physicochemical properties, high predicted potency, and good synthetic accessibility. Among the 212 candidates, 34 of them were manually selected and screened using miniaturized high-throughput experimentation (HTE), enabling rapid evaluation of their inhibitory activity against MAGL. This workflow led to the identification of 14 compounds that could be readily synthesized through simple alkylation reactions and endowed with an enhanced inhibitory activity on MAGL isoforms, including human, rat, mouse and macaque, compared to the initial hit compound. Notably, all the 14 compounds feature a central piperazine core or the bioisosteric 2-azaspiro [*3.3*]heptane motif. Two representative examples, the 2-azaspiro [*3.3*]heptane derivative **64** and the piperazine **65** (Figure 8), displayed remarkable potency, inhibiting *h*MAGL in the sub-nanomolar range with IC_50_ values of 0.1 and 0.6 nM, respectively. Compared with other analogues of this series, compounds **64** and **65** exhibited improved lipophilic efficiency (LipE), with values of 6.27 and 6.21, respectively, and demonstrated efficient cellular penetration, consistent with their ability to target the cytosolic enzyme MAGL. To further elucidate the binding mode, compound **64** was co-crystallized with *h*MAGL. The cyclopentyl substituent in *para* position of the pyridine nitrogen atom causes a flip of the pyridine moiety enabling additional hydrogen bond interaction with a water molecule and facilitating lipophilic contacts within a hydrophobic sub-pocket of the enzyme active site. The 3-(cyclopropyl-*1H*-1,2,4-triazol-1-*yl*)-2-azaspiro [*3.3*]heptane fragment forms key hydrogen bonds with Met123, Ala51 and Arg57, as well as with two water molecules, and establishes π-π stacking interactions with Tyr194. Finally, ABPP experiments confirmed the high selectivity of these compounds toward MAGL. Overall, this study highlights the potential of integrating miniaturized HTE and deep learning approaches to foster the discovery and optimization of potent MAGL inhibitors.

##### *N*-Benzyldienamides

In 2025, the research group led by Prof. Manzanares reported the identification of a novel MAGL inhibitor, MCH11 ((9*Z*,12*Z*)-*N*-benzyl-2-hydroxyoctadeca-9,12-dienamide, compound **66**, Figure 8), as a potential therapeutic agent for the treatment of alcohol use disorder (AUD) [139]. Notably, the original article neither reports the design or synthetic route of MCH11 (**66**), nor provides the IC_50_ value relative to enzymatic inhibitory activity on *h*MAGL. To date, such information has not been reported in other scientific publications or patent documents. In fact, in this study, the compound MCH11 (**66**) was commercially supplied by Medalchemy Laboratories (Alicante, Spain). Regarding its chemical structure, MCH11 (**66**) features a long alkyl chain composed of 17 carbon atoms bearing two double bonds, linked to a polar *N*-benzylacetamide group. This amphiphilic architecture closely resembles that of the endogenous MAGL substrate 2-AG. From a pharmacological point of view, MCH11 (**66**) was extensively characterized in vivo. The authors first assessed the pharmacokinetic profile of MCH11 (**66**) following acute intraperitoneal administration at different doses, demonstrating that the compound reached peak plasma concentrations approximately one hour after dosing and preferentially modulated 2-AG and related endocannabinoids. Behavioral evaluations revealed that MCH11 (**66**) produced anxiolytic- and antidepressant-like effects without inducing locomotor deficits or impairing cognitive performance, while also significantly reducing impulsivity. The compound was shown to markedly decrease ethanol consumption, preference, and motivation to drink in both voluntary ethanol consumption (VEC) and oral self-administration (OEA) models. Notably, MCH11 (**66**) displayed synergistic effects when co-administered with topiramate (TPO), an election drug used in clinic to manage AUD. Importantly, pronounced sex-dependent differences were observed across MCH11 (**66**) pharmacological effects, as the combined treatment with TPO displayed greater efficacy than either agent alone in male mice, whereas in female mice the improvement associated with the combination therapy was less pronounced. Collectively, these findings provide the first evidence that MCH11 (**66**) exerts anxiolytic and antidepressant-like effects while reducing the propensity to consume ethanol potentially through MAGL inhibition, supporting its potential as a promising pharmacological candidate for the treatment of AUD.

##### Esters

Very recently, Bononi and co-workers synthesized a small series of cetylated fatty acids to investigate their potential as MAGL inhibitors [140]. Cetylated fatty acids have widely been used in topical formulations for the treatment of musculoskeletal disorders, although the mechanism of action underlying their analgesic and anti-inflammatory effects has not yet been fully elucidated. Among the nine derivatives developed, the hexadecyl decanoate analogue **67** (Figure 8) emerged as the most potent inhibitor of this series showing the highest single-point inhibition (47.1%) when tested at 50 μM concentration. Owing to this promising result, a full dose–response study was performed, confirming the inhibitory activity of compound **67** on *h*MAGL with an IC_50_ value of 36 μM. Overall, these findings support the hypothesis that the analgesic and anti-inflammatory effects exerted by cetylated fatty acids may in part arise from MAGL inhibition.

## 3. Conclusions

MAGL has emerged over the last decade as a highly attractive pharmacological target due to its key regulatory role at the intersection between the ECS and lipid metabolism. By hydrolyzing 2-AG and, more generally, MAGs, MAGL is involved in several pathological processes, including inflammation, pain, neurodegeneration, metabolic disorders and cancer. Consequently, major research efforts have been directed toward the identification of selective MAGL modulators capable of finely tuning the activity of this enzyme.

As highlighted throughout this comprehensive review, the period from 2020 to the present has witnessed significant progress in the discovery and development of novel MAGL-targeting agents. A wide structural diversity of compounds has been reported, ranging from classical enzyme inhibitors to more innovative approaches (Section 2.1, Section 2.2 and Section 2.3), including the development of MAGL degraders, particularly anti-MAGL PROTAC **26**. All selective MAGL modulators discussed in this review are summarized in Table 1, providing an organized overview of their classification structural features, mechanisms of action and origin.

Our analysis of the literature indicates that the most recently developed irreversible MAGL inhibitors are predominantly based on fully synthetic small-molecule scaffolds (Section 2.1). A similar trend has been observed for recently developed reversible MAGL inhibitors, which are largely represented by piperidine- and piperazine-based derivatives, with only a few examples derived from natural sources (Section 2.2). In contrast, compounds with an undefined mechanism of MAGL inhibition appear to be more evenly distributed between natural and synthetic compounds (Section 2.3). This observation likely reflects the fact that many studies reporting MAGL inhibition primarily focus on the identification of bioactive compounds, while detailed mechanistic characterization is often performed at a subsequent stage. At the same time, these findings highlight the continued interest in natural products as valuable starting points for the development of new MAGL-targeting therapeutic strategies.

Notably, the class of reversible MAGL inhibitors appears to be more populated than that of irreversible inhibitors, reflecting the growing interest in reversible modulation of MAGL activity. This trend is driven by the increasing awareness of potential long-term effects associated with chronic MAGL blockade, which has prompted the identification of compounds capable of achieving a more controlled modulation of enzyme activity without affecting the ECS.

Importantly, recent years have also seen the emergence of innovative strategies aimed at modulating MAGL through mechanisms that go beyond classical enzyme inhibition (Section 2.1, Section 2.1.6. Miscellaneous). Approaches based on TPD, such as PROTAC technology, are opening new perspectives for both the pharmacological modulation and the biological investigation of MAGL. These strategies may provide complementary opportunities to better understand the complex physiological and pathological roles of this enzyme. In addition, increasing attention is being directed toward combinatorial therapeutic approaches involving MAGL modulation with the aim of simultaneously affecting lipid metabolic pathways and the tumor microenvironment [141].

Overall, the rapid expansion of the chemical space of MAGL modulators has been strongly supported by advances in medicinal chemistry, including machine learning and improved biochemical methods for assessing MAGL activity. Indeed, the integration of these multidisciplinary approaches plays a crucial role in the identification of next-generation MAGL modulators with improved potency, selectivity and pharmacological profiles.

Several emerging research directions may further broaden the therapeutic opportunities offered by targeting MAGL. For example, the identification of allosteric modulators of MAGL remains an unexplored research area that could expand the range of pharmacological strategies available for regulating this enzyme. Furthermore, growing interest is being directed toward multi-target strategies, as exemplified by the development of dual MAGL/FAAH inhibitors, with the aim of simultaneously modulating complementary pathways involved in endocannabinoid signaling and disease progression. Recent advances in artificial intelligence and machine learning are also beginning to impact MAGL drug discovery. For example, AI-assisted hit-to-lead optimization workflows integrating graph neural networks, reaction outcome prediction, potency estimation, and ADMET modeling have recently enabled the rapid identification of highly potent MAGL inhibitors, highlighting the potential of data-driven approaches to accelerate future ligand discovery and optimization [138].

From a translational perspective, clinical development of MAGL-targeting agents remains limited. Although the clinical evaluation of ABX-1431 (**14**) has demonstrated the feasibility of advancing MAGL inhibitors into human studies, the vast majority of MAGL modulators reported to date, including those discussed in this review, remain at the preclinical stage. This translational gap highlights the need for continued efforts to improve pharmacokinetic and safety profiles, together with a deeper mechanistic characterization of newly identified compounds, in order to facilitate their progression toward clinically relevant therapeutic strategies. Addressing these challenges will be essential to fully exploit the therapeutic potential of MAGL modulation.

Taken together, the studies analyzed in this review provide an exhaustive overview of the current landscape of MAGL modulators (2020–2026) and highlight promising directions for future research aimed at the rational design of novel agents targeting this important enzyme.

## Figures and Tables

**Table 1:** Summary of selective MAGL modulators developed from 2020 to 2026.

MAGL Modulators	Mechanism of Inhibition	Origin	Chemical Class	Compound	MAGL Inhibition or Degradation Activity	Selectivity Toward Other Serine Hydrolases	Ref
Inhibitors	Irreversible	Synthetic	Carbamates	**21**	IC_50_ = 1 µM	n.r.	[56]
			Disulfides	**22**	IC_50_ = 1.89 µM	FAAH: n.i.	[57]
			Ureas	(3*R*,4*S*)-**23**	IC_50_ = 0.021 nM	Off-targets: carboxyl esterases (CES), ABHD12, ABHD6 and LYPLA2 (inhibition values n.r.)	[60]
			Nitrile-based compounds	**24**	IC_50_ = 2.05 µM	n.r.	[61]
			Esters	**25**	IC_50_ = 21 µM	FAAH: IC_50_ > 100 µM	[63]
			PROTAC	JN-PROTAC (**26**)	DC_50_ = 60.28 nM	n.r.	[65]
	Reversible	Natural	Flavones	8-PN (**27**)	IC_50_ = 9.5 µM	n.r.	[69]
			Triterpenoids	NGR1 (**28**)	n.r.	n.r.	[74]
		Synthetic	Diazetidinyl diamides	**29**	IC_50_ < 5 nM	n.r.	[79]
				**30**		n.r.	
				**31**		FAAH: IC_50_ > 10 µM	
			Benzothiazolinones	**32**	IC_50_ = 34.1 nM	FAAH: IC_50_ = 9.38 µM ABHD6: IC_50_ = 3.50 µM	[80]
			Ureas	**33**	IC_50_ = 19.6 µM	FAAH: 82% activity at 25 µM	[83]
			Sulfonamides	**34**	IC_50_ = 34.7 µM	n.r.	[84]
			Piperidines and piperazines	**35**	IC_50_ = 14.75 nM	FAAH: IC_50_ > 100 µM	[85,86]
				**36**	IC_50_ = 10.3 nM	FAAH: IC_50_ > 10 µM	[87]
				DC630-8 (**37**)	IC_50_ = 2.84 µM	n.r.	[89]
				**38**	IC_50_ = 18 nM	FAAH, ABHD6 and ABHD12: IC_50_ > 10 µM	[90]
				**39**	IC_50_ = 1.26 nM	FAAH: IC_50_ > 20 µM	[91]
				**40**	IC_50_ = 2.0 nM	FAAH, ABHD6 and ABHD12: IC_50_ > 10 µM	[92]
				**41**	IC_50_ = 5.2 nM	FAAH, ABHD6 and ABHD12: IC_50_ > 10 µM	[94]
				**42**	IC_50_ = 14.6 µM	n.r.	[95]
				**43**	IC_50_ = 68.8 µM	FAAH: IC_50_ > 100 µM	[96]
				LEI-515 (**44**)	pIC_50_ = 9.3 (IC_50_ = 0.501 nM)	FAAH: n.i. ABHD6, ABHD12 and DAGL-α: >500-fold selectivity	[97,98]
				M-18C (**45**)	IC_50_ = 662.6 nM	n.r.	[99,100]
				**46**	pIC_50_ = 7.1 (IC_50_ = 79.4 nM)	ABHD6: <50% inhibition	[101]
				**47**	pIC_50_ = 8.0 (IC_50_ = 10 nM)	FAAH and ABHD6: n.i.	[102]
			Spiro carbamates	**48**	IC_50_ = 6.2 nM	FAAH and ABHD6: IC_50_ > 100 µM	[103,104]
			*cis*-Hexahydro-pyrido-oxazinones	**49**	IC_50_ = 32 nM	FAAH, ABHD6, ABHD12 and DAGL-α: IC_50_ > 10 µM	[105]
			Anilides	**50**	IC_50_ = 0.34 µM	FAAH: IC_50_ = 4.2 µM	[106]
	Undefined	Natural	Alkaloids	Dehydrocorydaline (DHC, **51**)	IC_50_ = 240.1 µM	n.r.	[107]
			γ-Lactones	Salinipostin N (**52**)	IC_50_ = 178 nM	n.r.	[108]
			Terpenes and terpenoids	Cryptotanshinone (**53**)	pIC_50_ = 4.9 (IC_50_ = 12.6 µM)	ABHD6 and ABHD12: IC_50_ > 100 µM	[109]
				β-Caryophyllene (BCP, **54**)	IC_50_ = 15.8 µM	n.r.	[112]
				Acetyl-11-keto-*β*-boswellic acid (AKBA, **55**)	n.r.	n.r.	[121]
			Phytosterols	*β*-sitosterol (**56**)	IC_50_ = 8.10 µg/mL (IC_50_ = 19.5 µM)	n.r.	[123]
			Glycosides	Cistanoside F (CF, **57**)	n.r.	n.r.	[124]
			Indolic derivatives	Melatonin (MLT, **58**)	n.r.	n.r.	[131]
		Synthetic	Benzimidazoles	Rabeprazole (**59**)	IC_50_ = 4.2 µM	n.r.	[132]
			Pyrrolidin-2-ones	**60**	IC_50_ = 9.4 nM	FAAH: IC_50_ > 50 µM	[133]
				**61**	IC_50_ = 7.6 nM	FAAH: IC_50_ = 68 µM	[134]
			Azaglycinamides	**62**	IC_50_ = 21.05 µM	n.r.	[136]
			Piperazines and their bioisosteres	Cetirizine (**63**)	IC_50_ = 9.39 µM	n.r.	[137]
				**64**	IC_50_ = 0.1 nM	Selective for MAGL (inhibition values n.r.)	[138]
				**65**	IC_50_ = 0.6 nM		
			*N*-Benzyldienamides	MCH11 (**66**)	n.r.	n.r.	[139]
			Esters	**67**	IC_50_ = 36 µM	n.r.	[140]

n.r. = not reported. n.i.= no inhibition.

## Data Availability

No new data were created or analyzed in this study. Data sharing is not applicable to this article.
